# Exploring the optimal strategy of imputation from SNP array to whole-genome sequencing data in farm animals

**DOI:** 10.3389/fgene.2022.963654

**Published:** 2022-08-26

**Authors:** Yifan Jiang, Hailiang Song, Hongding Gao, Qin Zhang, Xiangdong Ding

**Affiliations:** ^1^ National Engineering Laboratory for Animal Breeding, Laboratory of Animal Genetics, Breeding and Reproduction, Ministry of Agriculture and Rural Affairs, College of Animal Science and Technology, China Agricultural University, Beijing, China; ^2^ Beijing Key Laboratory of Fisheries Biotechnology, Fisheries Science Institute, Beijing Academy of Agriculture and Forestry Sciences, Beijing, China; ^3^ Natural Resources Institute Finland (Luke), Helsinki, Finland; ^4^ Shandong Provincial Key Laboratory of Animal Biotechnology and Disease Control and Prevention, Shandong Agricultural University, Taian, China

**Keywords:** imputation, accuracy, whole genome sequencing, livestock, poultry

## Abstract

Genotype imputation from BeadChip to whole-genome sequencing (WGS) data is a cost-effective method of obtaining genotypes of WGS variants. Beagle, one of the most popular imputation software programs, has been widely used for genotype inference in humans and non-human species. A few studies have systematically and comprehensively compared the performance of beagle versions and parameter settings of farm animals. Here, we investigated the imputation performance of three representative versions of Beagle (Beagle 4.1, Beagle 5.0, and Beagle 5.4), and the effective population size (Ne) parameter setting for three species (cattle, pig, and chicken). Six scenarios were investigated to explore the impact of certain key factors on imputation performance. The results showed that the default Ne (1,000,000) is not suitable for livestock and poultry in small reference or low-density arrays of target panels, with 2.47%–10.45% drops in accuracy. Beagle 5 significantly reduced the computation time (4.66-fold–13.24-fold) without an accuracy loss. In addition, using a large combined-reference panel or high-density chip provides greater imputation accuracy, especially for low minor allele frequency (MAF) variants. Finally, a highly significant correlation in the measures of imputation accuracy can be obtained with an MAF equal to or greater than 0.05.

## 1 Introduction

Genotype imputation (Yun et al., 2009), which uses linkage disequilibrium knowledge from haplotypes of a known reference panel to predict genotypes of missing or ungenotyped markers, is a commonly used procedure for obtaining more genotypes. This is achieved by imputing low-to high-density single nucleotide polymorphism (SNP) markers, and even whole-genome sequencing (WGS) SNP markers. It has played a crucial role in whole-genome studies such as genomic selection (GS) (Vanraden et al., 2017; Raymond et al., 2018; Zhang et al., 2018) and genome-wide association studies (GWAS) (Jonathan and Bryan, 2010; Kelemen et al., 2015; Yan et al., 2017; van den Berg et al., 2019). The availability of next-generation sequencing techniques has made it possible to obtain WGS and SNP markers at reasonable cost. However, sequencing all individuals is not realistic in livestock and poultry breeding programs. Thus, one of the most used strategies is to sequence a subset of a population that is used as a reference panel to perform genotype imputation with high accuracy. For example, using the comprehensive reference panels provided by the 1000 Genomes Project and 1000 Bull Genomes Project consortium to impute to whole-genome-level SNPs has recently become more common in humans and other genomic studies (Kelemen et al., 2015; Liu et al., 2015; Pausch et al., 2016).

Since its first release in 2009 (Browning and Browning, 2009), Beagle has been widely used for genotype imputation and phasing. Beagle uses Bayesian methods with the Markov Chain Monte Carlo (MCMC) algorithm. As one of the most popular imputation software programs, it has been widely used in humans and non-human species, such as cattle (Frischknecht et al., 2017), dogs (Jenkins et al., 2021), pigs (Yang et al., 2021; Li et al., 2022), and chickens (Ye et al., 2019a; Li et al., 2020b), etc. In the past 13 years, it has been continuously updated from Beagle 3 to Beagle 5.4 (as of 25 May 2022). Beagle 4.1 was developed for genotype imputation of millions of reference samples (Browning and Browning, 2016). Beagle 5.0 was developed to further reduce the computational cost of imputation from large reference panels (Browning et al., 2018). Since version 5.2, Beagle has employed a two-stage phasing algorithm to make it faster and more memory efficient (Browning et al., 2021). However, the differences of these version, and their effects of the parameter settings on livestock and poultry, have not been fully compared. Research have shown that the parameter effective population size (Ne) has the greatest impact on the error rate of imputation in chicken and maize populations (Pook et al., 2020). Thus, the effect of Ne on the imputation accuracy is considered in our study.

Factors affecting imputation accuracy, such as reference panel size and chip density, have already been studied based on both simulated and empirical data (Pausch et al., 2013; Ventura et al., 2016; Pausch et al., 2017). However, most of them were carried out with default parameters and were not intended to compare different imputation programs or parameter settings (Zheng et al., 2012; Pausch et al., 2013; Ventura et al., 2016), and the calculation of imputation accuracy is not similar between studies. For example, in some studies, only random masked sites were used for the calculation of imputation, and some used all imputed sites but only a part of the individuals (Frischknecht et al., 2017; Ye et al., 2018; Yuan et al., 2018). In addition, the commonly used measures of genotype imputation accuracy include genotype concordance, the correlation between imputed and true genotypes, and Allele R-Squared (AR2) and Dosage R-Squared (DR2) in different versions of Beagle (Pausch et al., 2017; Rowan et al., 2019; Song et al., 2019; van den Berg et al., 2019). Some studies only used one method to measure, which made the reliability of comparison between the studies low. Therefore, it is crucial to devise an optimal strategy for improving the accuracy of genotype imputation in GS and GWAS studies, or in livestock and poultry breeding programs, regardless of the chip density in the target panel. We performed a comprehensive and systematic investigation of these factors on imputation accuracy across three species: cattle, pigs, and chickens.

In the current study, we investigated the performance of three representative versions of Beagle (Beagle 4.1, Beagle 5.0, and Beagle 5.4) and the effects of parameter settings on three farm animals (cattle, pigs, and chickens) to devise an optimal strategy from the SNP array to whole genome sequencing data of livestock and poultry. In addition, we explored the effects of chip density, reference population size, and the relationship between the target panel and the reference panel on imputation accuracy. Finally, the correlation between the measures of imputation accuracy and minor allele frequency (MAF) was also explored.

## 2 Materials and methods

### 2.1 Whole genome sequencing data and BeadChip data

**FIGURE 1:**
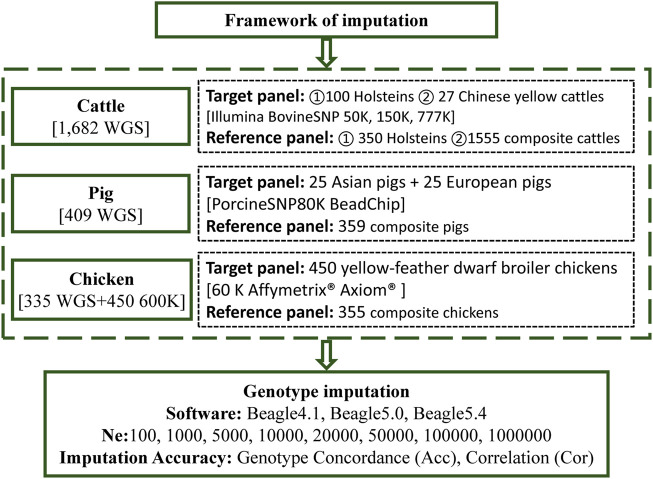
The framework of the imputation.

WGS and BeadChip data based on three livestock and poultry, including cattle, pigs, and chickens, were used in this study. The framework of the genotype imputation is shown in Figure 1. The detailed information is as follows.

#### 2.1.1 Cattle

WGS data, of Beagle-phased SNP calls, were obtained from RUN 5 of the 1000 Bull Genomes Project, released in 2017 (Daetwyler et al., 2014). A total of 1,682 whole-genome sequenced animals were provided by the 1000 Bull Genomes Project (Run 5), which included 1,602 *Bos taurus*, 53 *Bos indicus*, and 27 Chinese yellow cattle (Daetwyler et al., 2014). Detailed information regarding the breeds of the animals used is provided in Supplementary Table S1. A total of 67.33 million variants were discovered in these animals, of which 64.80 million were SNPs and 2.53 million were indels. Further details about variant calling, genotyping, and filtering of variants, in the 1000 Bull Genomes Project, were presented by Daetwyler et al. (2014).

Genotype imputation included two panels: the reference panel and the target panel. In the genotype imputation analysis scenarios we investigated, two main target panels and two main reference panels were considered. One of the target groups consisted of 100 Holstein cattle, randomly selected from the 450 Holstein cattle in the sequencing data, and the other consisted of 27 Chinese yellow cattle. Correspondingly, the remaining 350 Holsteins served as the reference panel of purebreds (ref350). All the remaining 1,555 cattle in the 1000 Bull Genomes Project, RUN5, served as a composite reference panel (ref1555).

To investigate the influence of different imputation scenarios, the genotypes of the target panel were masked, using bovine chips of different densities, to mimic the scenario from which the animals were genotyped. The low-, medium-, and high-density chips corresponded to Illumina BovineSNP 50, 150, and 777 K BeadChip chips, of cattle with 54,609, 138,892, and 777,962 SNPs, respectively. In addition, the positions of all SNPs, in BeadChip, were based on the *B. taurus* UMD3. We used one reference genome (Zimin et al., 2009) obtained from the UCSC liftover (http://genome.ucsc.edu/cgi-bin/hgLiftOver), which was consistent with a genome of the 1000 Bull Genomes Project RUN5. After removing variants with minor allele counts (less than one), and variants with more than two alleles across all reference individuals, Table 1 presents detailed information on variants in the imputation. Then, imputation from chip variants to whole genome sequence variants was performed and the imputation accuracy of imputation (IMP) sites of the target population was compared to the WGS data of these target individuals.

**TABLE 1:** Number of SNPs used across chromosomes under different panels in cattle.

Chr (Cattle)	Chr length (bp)	Reference panel		Target panel			IMP sites (ref350)			IMP sites (ref1555)		
		ref350	ref1555	50 K	150 K	777 K	50 K	150 K	777 K	50 K	150 K	777 K
chr1	158,337,067	1,265,065	3,068,377	3,067	6,781	39,186	1,262,134	1,258,329	1,229,993	3,065,312	3,061,599	3,029,193
chr7	112,638,659	847,075	2,063,921	2,064	5,386	28,133	845,108	841,843	822,367	2,061,858	2,058,537	2,035,791
chr21	71,599,096	562,318	1,387,487	1,296	3,071	17,712	561,083	559,274	546,263	1,386,192	1,384,416	1,369,777
chr29	51,505,224	470,173	1,101,854	962	2,190	12,038	469,260	467,998	458,834	1,100,892	1,099,665	1,089,816
Total	394,080,046	3,144,631	7,621,639	7,389	17,428	97,069	3,137,585	3,127,444	3,057,457	7,614,254	7,604,217	7,524,577

Chr, chromosome; IMP sites, imputed sites and locus used to calculate imputation accuracy.

#### 2.1.2 Pig

WGS data for pigs were downloaded from the Genome Variation Map (GVM; http://bigd.big.ac.cn/gvm/) database, which collected and integrated genome variations for 47 species (as of 25 May 2022) (Li et al., 2020a). A total of 409 pigs, with 90.90 million SNPs (based on the *Sus scrofa* 10.2 reference genome), were provided by GVM, which included 213 Asian pigs, 181 European pigs, and 15 *Sus* pig species (Supplementary Table S2). Variants with a missing rate of more than 0.2, and a minimum allele frequency of less than 0.01, were removed for subsequent analysis. Phasing was executed using Beagle (version5.4) (Browning et al., 2021), with its default parameters. We randomly selected 25 European and 25 Asian pigs, as the target population and the remaining 359 pigs were a part of the reference population. The genotypes of the target panel were masked to a PorcineSNP80K BeadChip (Illumina, San Diego, CA, United States). After imputation, like that of cattle, the imputation accuracy was calculated by comparing the IMP sites in the target population with the WGS data of target individuals. The statistics of the number of SNPs are listed in Table 2.

**TABLE 2:** Number of SNPs used across chromosomes in pigs and chickens.

Species	Chr	Chr length (bp)	Reference panel	Target panel	IMP sites
Pig	chr1	315,321,322	2,756,826	5,014	2,751,812
	chr6	157,765,593	1,782,136	3,693	1,778,443
	chr12	63,588,571	893,925	2,138	891,787
	chr18	61,220,071	879,515	1,439	878,076
	Total	597,895,557	6,312,402	12,284	6,300,118
Chicken	chr1	196,202,544	7,158,664	9,841	80,339
	chr3	111,302,122	4,079,325	5,506	44,859
	chr6	35,467,016	1,479,613	2,117	17,537
	chr28	4,974,273	190,787	534	4,187
	Total	347,945,955	12,908,389	17,998	146,922

Chr, chromosome; IMP sites, imputed sites and locus used to calculate imputation accuracy.

#### 2.1.3 Chicken

This dataset was adopted from Ye et al.’s studies (Ye et al., 2018; Yuan et al., 2018). A total of 335 chickens were sequenced using WGS technology (based on the galGal5 reference genome), and 450 yellow-feather dwarf broiler chickens were genotyped using the 600 K Affymetrix^®^ Axiom^®^ high-density genotyping array (Supplementary Table S3). The WGS panel contains diverse breeds including red junglefowl, green junglefowl, Tibetan chickens, fighting chickens, white leghorn chickens and so on. It is worth mentioning that 24 key individuals of the yellow-feather dwarf broiler population were included in the 355 WGS populations. Following Ye et al. (2018), Ye et al. (2019b), the supposed 60 K chip data were generated by sampling the first SNP in each bin of adjacent 10 SNPs, of the 600 K SNP chip as the target panel for imputation. The 450 chickens with a 60 K BeadChip chip were used as the target panel, and the 335 WGS chickens were used as the reference panel for imputation. After the imputation was performed, the IMP sites coincident with 600 K were used to calculate the imputation accuracy, as shown in Table 2.

### 2.2 Genotype imputation strategy

To improve computational efficiency, four autosomes across large, medium, and small chromosomes, were separately selected for cattle (chr1, chr7, chr21, chr29), pig (chr1, chr6, chr12, chr18), and chicken (chr1, chr3, chr6, chr28). The variant information of the genotype imputation in this study is listed in Tables 1 and 2.

We compared the effect of Beagle versions, setting effective population size (Ne), chip density, reference panel sizing, and the relationship between the target and reference panels on imputation accuracy, as shown in Table 3. To explore the effect of the Beagle version and the parameter of effective population size (Ne) on the imputation accuracy of the three livestock and poultry, the imputation were performed by Beagle 4.1 (Beagle.27Jan18.7e1.jar) (Browning and Browning, 2016), Beagle 5.0 (beagle.12Jul19.0df.jar) (Browning et al., 2018), and Beagle 5.4 (beagle.19Apr22.7c0.jar) (Browning et al., 2021) with the parameters of effective population size (Ne) set to 100, 1,000, 5,000, 10,000, 20,000, 50,000, 100,000, and 1,000,000 for the three livestock. In both Beagle 4.1 and Beagle 5.0, the default parameter of Ne was 1,000,000, but in Beagle 5.4, the default parameter of Ne was 100,000. Furthermore, in cattle populations, the effects of reference population size and chip density on imputation accuracy were also explored. Furthermore, using cattle as an example, we explored the relationship between the target and reference panels on the accuracy of genotype imputation with 27 Chinese yellow cattle as the target panel. Meanwhile, the imputation accuracy against minor allele frequency, the correlation of the measure of imputation accuracy, and the time used was explored using cattle datasets.

**TABLE 3:** Scenarios used to evaluate imputation performance.

Scenario	Description	Species	Target panel	Reference panel	Software	Ne
S1	Effects of beagle version and Ne parameter size on imputation accuracy in three species	Cattle	100 Holstein (50, 150, 777 K)	ref350, ref1555	Beagle4.1, Beagle5.0, Beagle5.4	100, 1,000, 5,000, 10,000, 20,000, 50,000, 100,000, 1,000,000
		Pig	25 Asian pigs + 25 European pigs (80 K)	359 pigs	Beagle4.1, Beagle5.0, Beagle5.4	100, 1,000, 5,000, 10,000, 20,000, 50,000, 100,000, 1,000,000
		Chicken	450 yellow-feather dwarf broiler chickens (60 K)	355 chickens	Beagle4.1, Beagle5.0, Beagle5.4	100, 1,000, 5,000, 10,000, 20,000, 50,000, 100,000, 1,000,000
S2	Chip density and reference panel size on the imputation accuracy	Cattle	100 Holstein (50, 150, 777 K)	ref350, ref1555	Beagle4.1, Beagle5.0, Beagle5.4	100,000
S3	Imputation accuracy against minor allele frequency	Cattle	100 Holstein (50, 150, 777 K)	ref350, ref1555	Beagle5.4	100,000
S4	The relationship of the measure of imputation accuracy (Acc, Cor, AR2, DR2)	Cattle	100 Holstein (50, 150, 777 K)	ref350, ref1555	Beagle4.1 (for AR2), Beagle5.4	100,000
S5	The relationship between target panel and reference panel on the imputation accuracy	Cattle	27 Chinese yellow cattle (50, 150, 777 K) and 100 Holstein (50, 150, 777 K)	ref350, ref1555	Beagle5.4	100,000
S6	Time consuming	Cattle	100 Holstein (50, 150, 777 K)	ref350, ref1555	Beagle4.1, Beagle5.0, Beagle5.4	100,000

Ne, effective population size; AR2, allelic R-squared; DR2, dosage R-squared; Acc, genotype concordance; Cor: correlation.

### 2.3 Evaluation of imputation accuracy

Two criteria were used to measure the imputation performance: 1) correlation between true and imputed genotypes (Cor), which were coded as 0, 1, and 2 for genotypes AA, AB, and BB, respectively; 2) genotype concordance (Acc), which was defined as the proportion of genotypes of the imputed variants that were the same as the true genotypes. In addition, Allele R-Squared (AR2, estimated squared correlation between the most probable REF dose and true REF dose) and Dosage-R2 (DR2, estimated squared correlation between estimated REF dose and true REF dose) output by Beagle (Beagle 4.1 generates both the AR2 and DR2, Beagle 5 only generates DR2) were also used to make a comparison of these imputation accuracy measurements.

### 2.4 Population structural analysis

The population structure was demonstrated by principal component analysis (PCA), using GCTA (version 1.92.0 beta2) software (Yang et al., 2011), and the first 20 eigenvectors were output and then plotted using the R program (Valero-Mora, 2010). Variants with an MAF of less than 0.05 were removed for this analysis.

## 3 Results

### 3.1 Population structure

**FIGURE 2:**
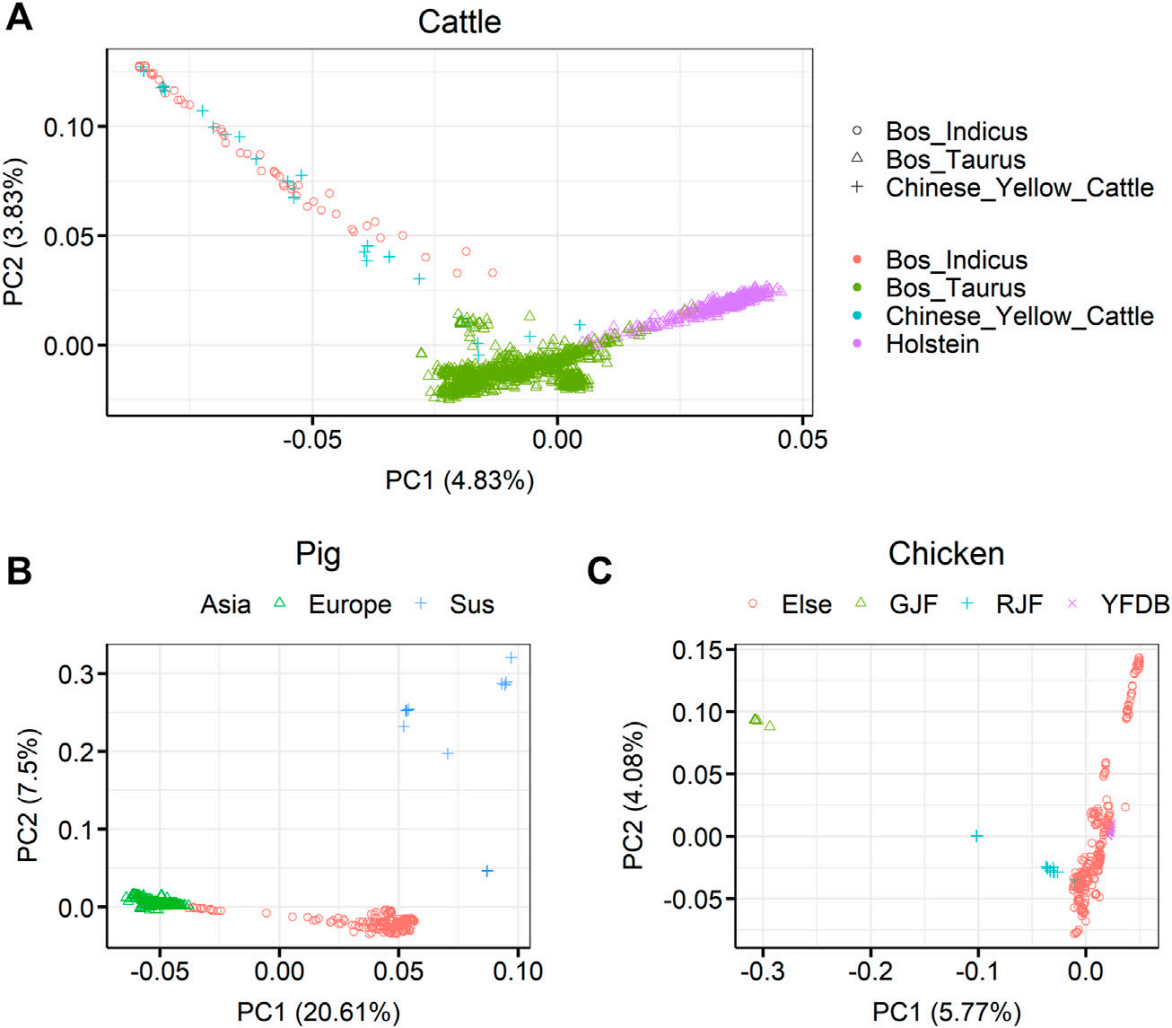
Principal component analysis (PCA) showing the population structure of the three farm animals (cattle, pigs, and chickens). **(A)** PCA showing the population structure of 1,682 sequenced cattle in the RUN5 of the 1000 bull genome project. **(B)** PCA showing the population structure of 409 sequenced pigs in genome variation map database **(C)** PCA showing the population structure of 335 sequenced chickens. GJF, green jungle fowl; RJF, red jungle fowl; YFDB, yellow feather dwarf broiler. Different colors and symbols represent different classes.

Principal component analysis (PCA) for the three livestock, cattle, pig, and chicken, is shown in Figure 2. For cattle, it can be seen that *Bos taurus* and *Bos indicus* were first separated by PC1 in 1,682 individuals, and then the individuals were separated into *B. taurus*, *B. indicus*, or Chinese yellow cattle by PC2. Among the *B. taurus*, Holstein cattle had the largest number of individuals were Holstein cattle (450 samples). For pigs, it was clearly shown that European pigs, Asian pigs, and *Sus* species pigs were separate from 409 pigs. For chickens, it was clearly shown that red jungle fowl and green jungle fowl separate from the other samples in 335 chickens. The detailed breed compositions are presented in Supplementary Tables S1–S3.

### 3.2 Beagle versions and the parameter of the effective population size settings on imputation accuracy

**FIGURE 3:**
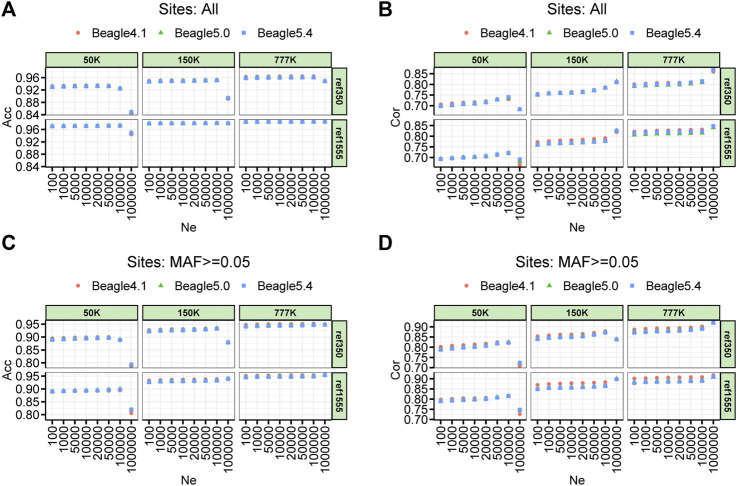
Accuracy of imputation for three density BeadChip chips, two reference population sizes and three imputation software with a range of effective population size (Ne) sets in cattle. **(A)** Imputation accuracy measured by the genotype concordance (Acc). **(B)** Imputation accuracy measured by the correlation (Cor) **(C,D)** corresponds to **(A)** and **(B)** with minor allele frequency sites less than 0.05 removed.

The imputation accuracy of the parameter setting on the effective population size (Ne) of three different Beagle versions for cattle, pigs, and chickens are shown in Figure 3 and Supplementary Figures S1, S2, respectively. For the comparison of imputation accuracy of the different versions of Beagle, we found that the three versions of Beagle software achieved almost the same accuracy in different scenarios with only slight differences. Beagle 5.0 and Beagle 5.4 performed nearly the same imputation accuracy across all scenarios. Compared with Beagle 4.1, Beagle 5 (including Beagle 5.0 and Beagle 5.4) showed 0.1% and 0.6% improvement in Acc, and 0.4% and 0.9% improvement in Cor for pigs and chickens, respectively, when Ne was equal to 100,000 (Supplementary Figures S1, S2). Similarly, the imputation accuracy varies by a few tenths of thousands of beagles. However, the size of Ne has a significant impact on imputation accuracy. In the case where the default Ne size of Beagle 4.1 and Beagle 5.0 (Ne = 1,000,000), the imputation accuracy for the cattle’s 50 K was significantly reduced, whether imputed with ref350 (Acc and Cor dropped by 7.72% and 5.28%, respectively, for all imputed sites; Acc and Cor dropped by 9.77% and 10.45%, respectively, for the imputed sites with MAF ≥ 0.05) or ref1555 (Acc and Cor dropped by 2.47% and 4.13%, respectively, for all imputed sites; Acc and Cor dropped by 8.24% and 7.55%, respectively, for the imputed sites with MAF ≥ 0.05). The imputation accuracy also decreased when the imputation was performed from the 150 K chip to the WGS, with ref350 (Acc dropped by 5.88% and no drop in Cor for all imputed sites; Acc and Cor dropped by 5.40% and 3.62%, respectively, for the imputed sites with MAF ≥ 0.05). The other panels in cattle imputation, such as imputation from 777 K to WGS and from 150 K to WGS with ref350, Ne had less impact on the accuracy of imputation (Figure 3). In addition, we noticed that in pig and chicken imputation, the default Ne size in Beagle 4.1 and Beagle 5.0 (Ne = 1,000,000) also reduced the imputation accuracy (Supplementary Figures S1, S2). All these results suggest that the impact of different Beagle versions on the imputation accuracy is small, but the default value of Ne has a great impact on the imputation accuracy, especially for the imputation of low-density chips or small reference panels.

### 3.3 Impact of reference population size and chip density on imputation accuracy

The imputation accuracy when using different chip densities with different reference population sizes is shown in Figure 3. Overall, the large reference populations resulted in higher imputation accuracy. The imputation accuracies measured by genotype concordances (Acc) at 50, 150, and 777 K, using ref350, were 0.925, 0.952, and 0.962, respectively, and their corresponding Cor values were 0.735, 0.784, and 0.812, respectively. The imputation accuracies measured by Acc at 50, 150, and 777 K, using ref1555, were 0.972, 0.981, and 0.985, respectively, and their corresponding Cor values were 0.720, 0.781, and 0.823, respectively. In general, the higher the chip density of the target panel, and the larger the number of reference panels, the higher the imputation accuracy. The Acc at chip densities of 50, 150, and 777 K were improved by 4.73%, 2.88%, and 2.30%, respectively, when the reference population was increased from ref350 to ref1555.

### 3.4 Effect of minor allele frequency on the imputation accuracy

**FIGURE 4:**
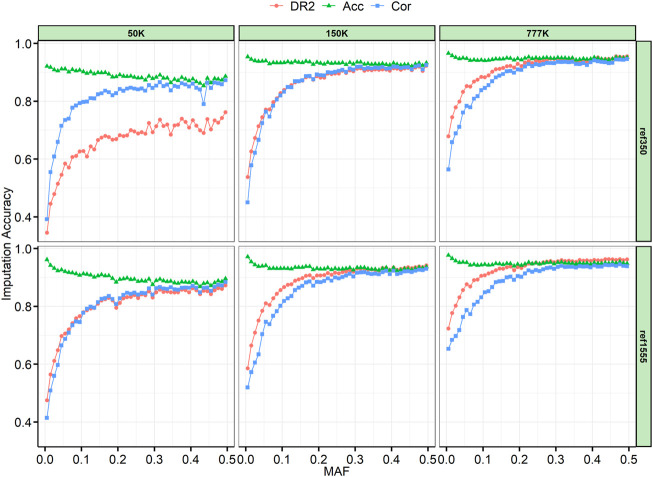
Imputation accuracy by minor allele frequency (MAF) class. The SNPs were divided into bins of 0.01 per increment according to their MAF. AR2, allelic R-squared; DR2, dosage R-squared; Acc, genotype concordance; Cor, correlation.

The SNPs were divided into 50 successive bins according to their MAF, with 0.01 step increments. Generally, we found that Acc was slightly decreased when Cor and DR2 were high (Figure 4). Because AR2 is only generated by Beagle 4.1, we also provide an example of the imputation results from 150 K to the two reference panels, which are like the results in Supplementary Figure S3, and AR2 is slightly lower than DR2. As expected, the imputation accuracy increased with an increase in MAF, and the accuracy changed rapidly when the MAF was less than 0.05. In addition, we can see that with a large reference panel, the imputation accuracy of low-MAF sites can be significantly improved. When the reference panel was increased from ref350 to ref1555, the lowest classified MAF site bin (MAF ≤ 0.01) imputation accuracy of Acc for 50, 150, and 777 K chips increased by 4.05%, 1.78%, and 1.16%, respectively; Cor increased by 2.28%, 6.97%, and 8.93%, and DR2 increased by 13.05%, 4.81%, and 4.52%, respectively. In the case of the same reference panel, with the increase in chip density, the imputation accuracy of the low-MAF sites will also be greatly improved. When the chip density was increased from 50 to 777 K, the imputation accuracy of Acc, Cor, and DR2 for ref350 increased by 4.44%, 17.21%, and 33.32%, respectively, and by 1.54%, 23.86%, and 24.80%, respectively, for ref1555.

### 3.5 The correlation between different measures of imputation accuracy

**FIGURE 5:**
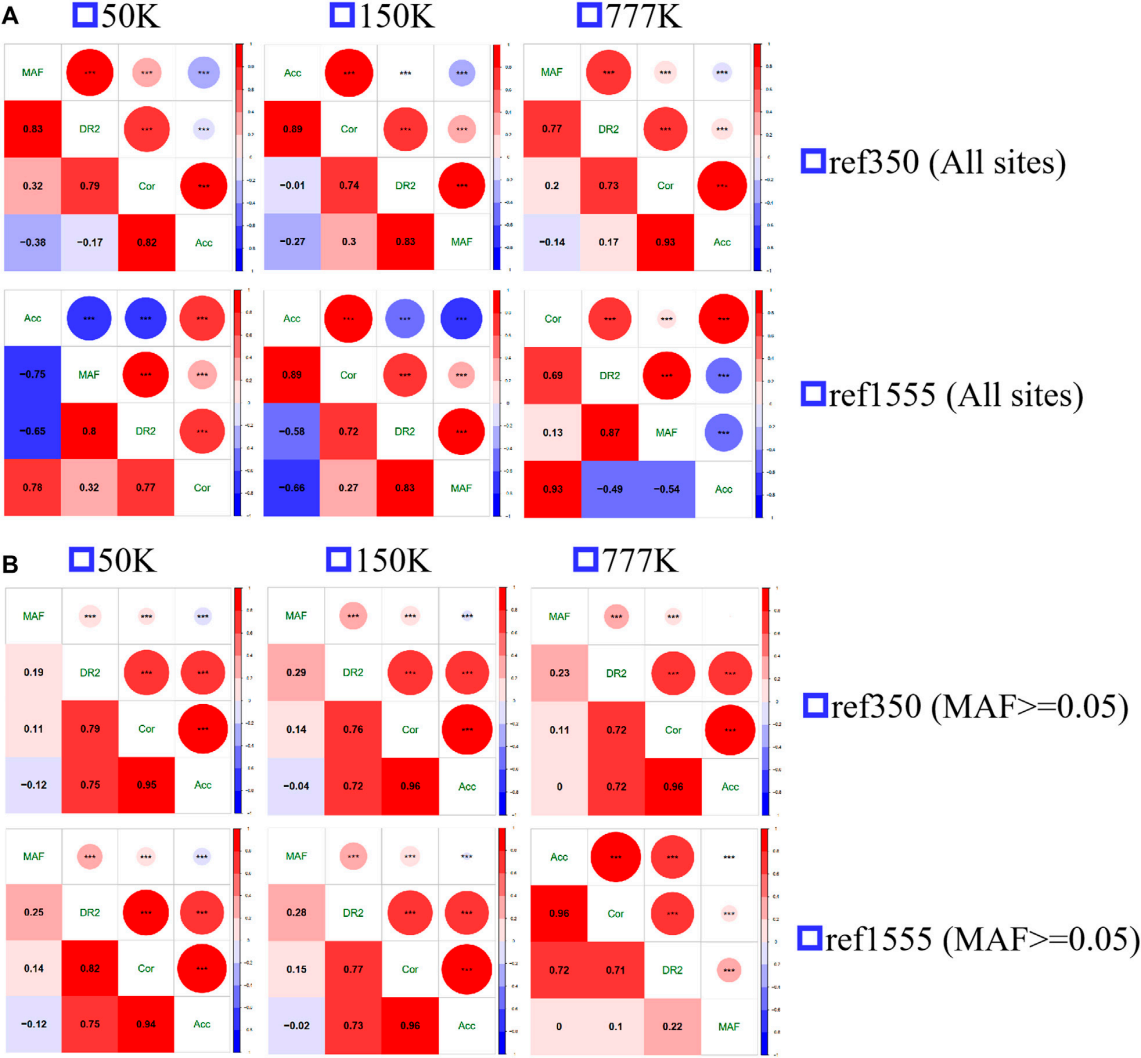
The spearman correlation of the three measures of imputation accuracy and minor allele frequency (MAF) among each other. **(A)** All sites **(B)** the sites with minor allele frequency no less than 0.05.

The Spearman correlation between MAF and the three measures of imputation accuracy was calculated and plotted, as shown in Figure 5. All the correlations were significant, with strong positive correlations between Acc and Cor (range from 0.78 to 0.93 with an average of 0.87), Cor and DR2 (range from 0.69 to 0.79, with an average of 0.74) at all loci, and Acc was moderately negatively correlated with MAF (range from −0.38 to −0.14, with an average of −0.26 for ref350, ranging from −0.75 to −0.53, with an average of −0.65 for ref1555), while DR2 had a strong positive correlation with MAF (range from 0.77 to 0.87, with an average of 0.83). Since the inconsistency between Acc and other accuracy measures was mainly in the case of MAF < 0.05 (Figure 4; Supplementary Figure S3), we also calculated the correlation after removing the sites with MAF less than 0.05. Here, we found a strong positive correlation between Acc, Cor, and DR2, with Acc and Cor being 0.96, Acc and DR2 being 0.73, and Cor and DR2 being 0.76. There was a weak negative correlation between Acc and MAF (−0.05), and a weak positive correlation between Cor and MAF (0.12), DR2, and MAF (0.24). Similarly, we also evaluated the correlation of AR2 with other metrics using imputation from 150 K to the two reference panels. As expected, there is a high correlation between AR2 and DR2 (0.98 for all sites and 1 for the sites with MAF greater than or equal to 0.05) (Supplementary Figure S4), which may be the reason why only DR2, and no AR2, output was observed after subsequent Beagle 5.0 version analysis.

### 3.6 The relationship between target and reference individuals on the imputation accuracy

**FIGURE 6:**
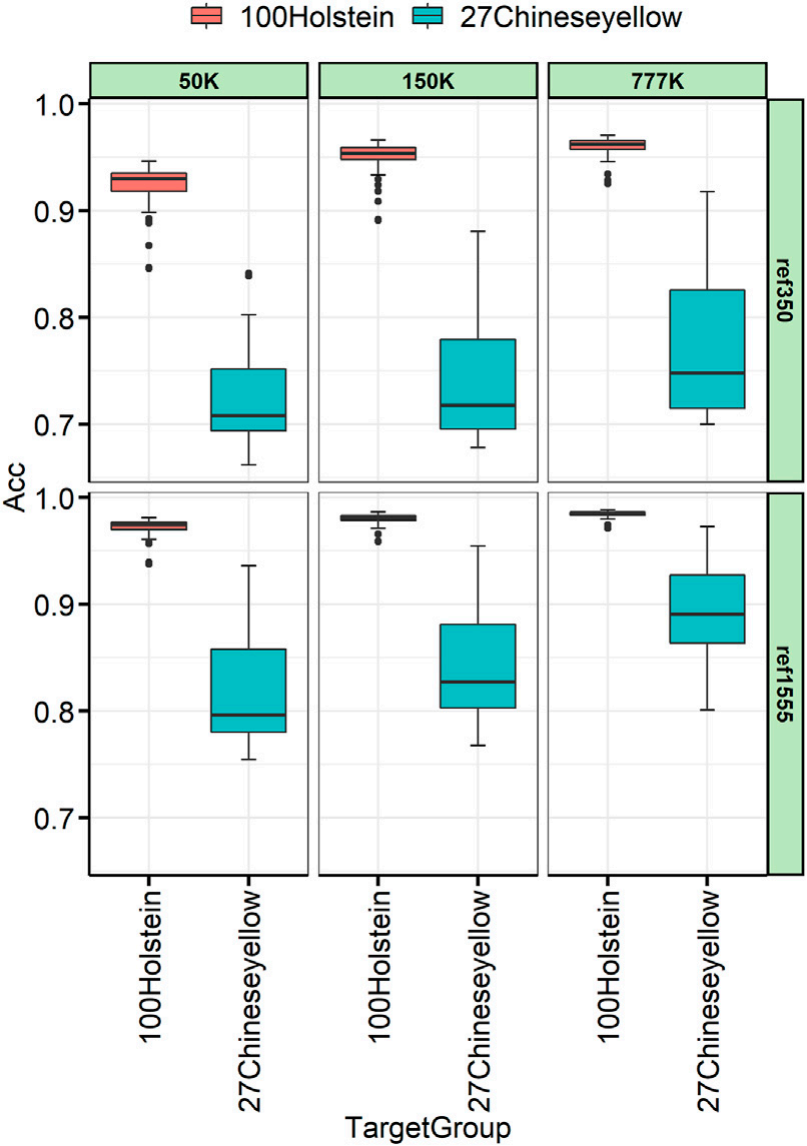
Genotype concordance calculated in the individual lever for 100 Holstein and 27 Chinese yellow cattle.

To better understand the relationship between the target and the reference individuals, 27 Chinese yellow cattle were used as the target panel for imputation, which had a complex history between *B. taurus* and *indicus*. The imputation accuracy varies across individuals, as shown in Figure 6. The variance among Chinese yellow cattle was much larger than that among the Holstein target individuals. Taking the imputation from 150 K to WGS, with ref1555, as an example, the imputation accuracies ranged from 0.768 to 0.953, and Mongolian cattle achieved the highest imputation accuracy, followed by Yanbian, Hasake, and Xizang cattle, which were 0.953, 0.941, 0.919, and 0.888, respectively. Nanyang cattle achieved the lowest imputation accuracy, followed by Liping, which was 0.768 and 0.789, respectively, as shown in Table 4. Other breeds such as Qinchuan, Luxi, Guanling, Dengchuan, Wenling, Dabieshan, and Fujian ranged from 0.802 to 0.871.

**TABLE 4:** The imputation accuracy for 27 Chinese yellow cattle.

Breed	Individual number	Imputation accuracy
Menggu	2	0.953
Yanbian	2	0.941
Hasake	2	0.919
Xizang	1	0.888
Qinchuan	2	0.871
Luxi	2	0.869
Guanling	2	0.837
Dengchuan	2	0.832
Wenling	2	0.808
Dehong	2	0.805
Dabieshan	2	0.802
Fujian	2	0.802
Liping	2	0.789
Nanyang	2	0.768

Imputation accuracy was measured using genotype concordance (Acc). This imputation was performed from 150 K to WGS with ref1555 using Beagle 5.2 with Ne = 1,00,000.

### 3.7 Running time

**FIGURE 7:**
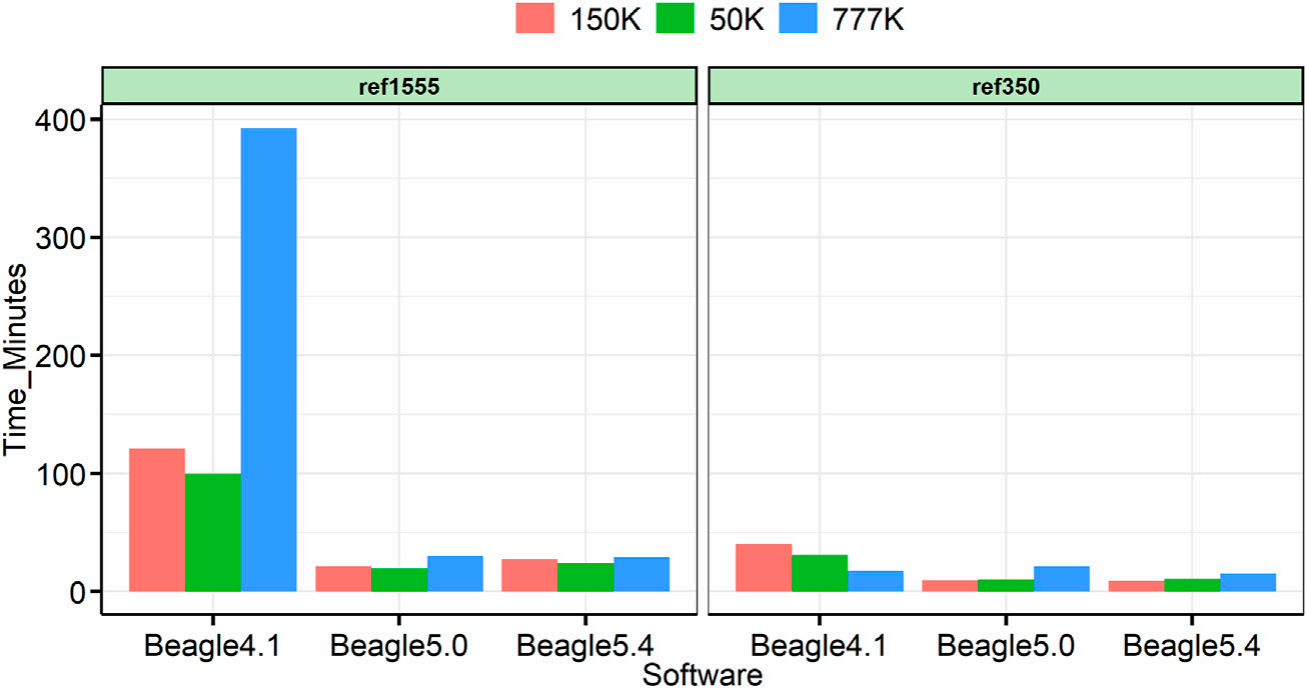
Time utilized for each imputation.

All analyses were run on a 22-core 2.10 GHz Linux computer, with Intel(R) Xeon(R) Gold 6,238 processors, and 1,007 GB of memory. Beagle was run on 24 threads. Figure 7 shows the computation time for each panel of cattle. In all cases, Beagle 5 is significantly faster than Beagle 4.1, and Beagle 5.0 is comparable to Beagle 5.4. In many reference panels, the obvious advantages of Beagle 5 can be obtained at 4.6-fold, 5.0-fold, and 13.2-fold, faster than Beagle 4.1 for the imputation of 50, 150, and 777 K, respectively.

## 4 Discussion

Imputation has been widely adopted, in the genomic era, as an important approach to boost the power of genetic studies of animal and human traits. By using the genotypes obtained from the 1000 Bull Genomes Project as the benchmark, with the incorporation of pig datasets from the GVM database, as well as the chicken datasets (Yuan et al., 2018), we systematically assessed the imputation performance of three representative versions of Beagle software with sets of effective population size across the three livestock and poultry. We also identified the influence of several key factors on imputation accuracy, such as chip density, the size of the reference panel, the relationship between the target panel and the reference panel, and the correlation between the measures of accuracy and the MAF. Overall, these key factors must be considered before performing an imputation.

With the continuous update of Beagle versions, various versions of the Beagle software were used in the published research, and the vast majority of studies used the default parameters (Li et al., 2020b; Li et al., 2022). However, in our study, we discovered that it is not suitable to use the default parameter Ne (default Ne = 1,000,000 for Beagle4.1 and Beagle5.0) when the number of reference panels is small or the chip density of the target panel is low, which will drop sharply, with drops ranging from 2.47% to 10.45% under our imputation cases (Figure 3). A similar result was reported by Pook et al. (2020) for the maize population, the imputation error rate increased when using default parameters in Beagle. It is worth noting that the default size of Ne in Beagle’s latest version, 5.4, is 100,000; in this case, all three versions of Beagle can obtain high imputation accuracy. This reminds us that it is more appropriate to set Ne to 100,000 to obtain higher accuracy because there is no reference panel as large as cattle, in other livestock and poultry (Supplementary Figures S1, S2). All these results indicate that the default Ne parameters are better changed when using earlier Beagle versions. Furthermore, there is little difference in imputation accuracy among the three versions (only thousandths of the change), but Beagle 5 can significantly sped up the computation, especially with large reference panels (13.24-fold faster in our cases) (Figure 7).

Our results showed that using a large mixed-breed reference population attained a much higher imputation accuracy than using a small single-breed reference population of the same breed as the target population, which is in agreement with the studies of Brøndum et al. (2014), Pausch et al. (2017). The reason for this high imputation accuracy for large mixed reference panels may be the variety of haplotypes in the reference panel, and their ability to facilitate the identification of long-shared haplotypes. With the development of next-generation sequencing technology, sequencing has decreased by five orders of magnitude (Smith, 1993), and the size of data sets used as reference panels for genotype imputation has increased rapidly. Especially for the genome projects implemented, such as the Human Project (Michael, 2005; Adam et al., 2015), the 1000 Bull Genomes Project (Daetwyler et al., 2014), and the dog genome projects (https://www.broadinstitute.org/scientific-community/science/projects/mammals-models/dog/dog-genome-links), which greatly facilitated imputation.

Previous studies have suggested that a low allele frequency may play an important role in complex traits (Manolio et al., 2009). However, it is challenging to correct the imputation of variants at low MAF and rare variants. Similar to previous studies (Teng et al., 2022), we also found that the accuracy dropped sharply for variants with MAF less than 0.05. In agreement with published research (Brøndum et al., 2014; Pausch et al., 2017), a multibreed combined reference panel increased imputation accuracy at low MAF variants. In addition, we found that the increase in chip density and imputation accuracy could also be improved at low MAF variants (Figure 4).

Across studies, there are different measures to evaluate the accuracy of imputation (Pausch et al., 2017; Yan et al., 2017; Song et al., 2019), including the genotype concordance, which counts the proportion of the correctly imputed sites to all imputed sites (Acc) and it is equal to 1 minus imputation error rates (the number of incorrectly imputed sites), the Pearson correlation between true and imputed genotypes (Cor), allelic R-squared (AR2, estimated squared correlation between the most probable REF dose and true REF dose), and Dosage R-Squared (DR2, estimated squared correlation between estimated REF dose and true REF dose) proposed in Beagle. The calculation of Acc and Cor requires the true genotype value, which is generally used to compare imputation methods. AR2 and DR2 are output by Beagle and are proposed as useful measures of imputation accuracy, usually used without knowledge of the true genotype information of the individuals belonging to the target panel. Our results indicated a significantly high correlation between AR2 and DR2 (Supplementary Figure S4), which may explain why only DR2 was the output after the Beagle 5 version. After removing the variants with MAF less than 0.05, a significantly high correlation was observed among the measures of accuracy, as well as a low correlation between MAF. This suggests that one of the metrics may be sufficient to measure the imputation accuracy.

For the imputation from low-density Beadchip to whole genome sequence variants, there are two approaches, one is the one-step imputation, referred direct imputed from low-density chip to WGS, the other is two-step imputation approach, referred imputed from low-density Beadchip to high-density Beadchip at first, and then impute to WGS. Part of the previous studies showed that the two-step imputation suggested to be advantageous in comparison to the one-step imputation approach with regard to imputation accuracy (Binsbergen et al., 2014). However, it had also been shown that the one-step imputation method yields higher imputation accuracy compared to the two-step imputation when fewer animals are available in the intermediate imputation steps (Korkuć et al., 2019). And the two-step imputation is difficult to implement in animals other than cattle since the need for high-density chip populations in large number individuals, and can be affected by the population structure of the high density mediated population. Thus, only one-step imputation was concerned in this study.

## 5 Conclusion

In summary, this study investigated the performance of three representative versions of Beagle (Beagle 4.1, Beagle 5.0, and Beagle 5.4) and the effects of parameter settings on three livestock and poultry (cattle, pig, and chicken) breeds. We found that the default parameter Ne, for the earlier version of Beagle, is not suitable for livestock and poultry in small reference panels or low-density BeadChip chips of target panels. Beagle 5 significantly reduced the computation time without a loss of accuracy, especially for large reference panels. Overall, a large, combined reference panel, or high-density chip, provided greater imputation accuracy, particularly for low minor allele frequency variants. Furthermore, AR2 or DR2 can be used to measure imputation accuracy in the absence of a true genotype. Our findings provide insights into the imputation from BeadChip data to whole-genome sequence variants of livestock and poultry, as well as other non-human species.

## Data Availability

Publicly available datasets were analyzed in this study. The names of the repository/repositories and accession number(s) can be found in the article/Supplementary Material.
