# Supplementary material for: Exploring the optimal strategy of imputation from SNP array to whole-genome sequencing data in farm animals
Source: Front Genet. 2022 Aug 26;13:963654. doi: 10.3389/fgene.2022.963654 (PMC9459117; doi:10.3389/fgene.2022.963654)
Supplement: Supplementary file 1 [file DataSheet1.docx]

Supplementary Material

# Supplementary Figures and Tables

## Supplementary Figures


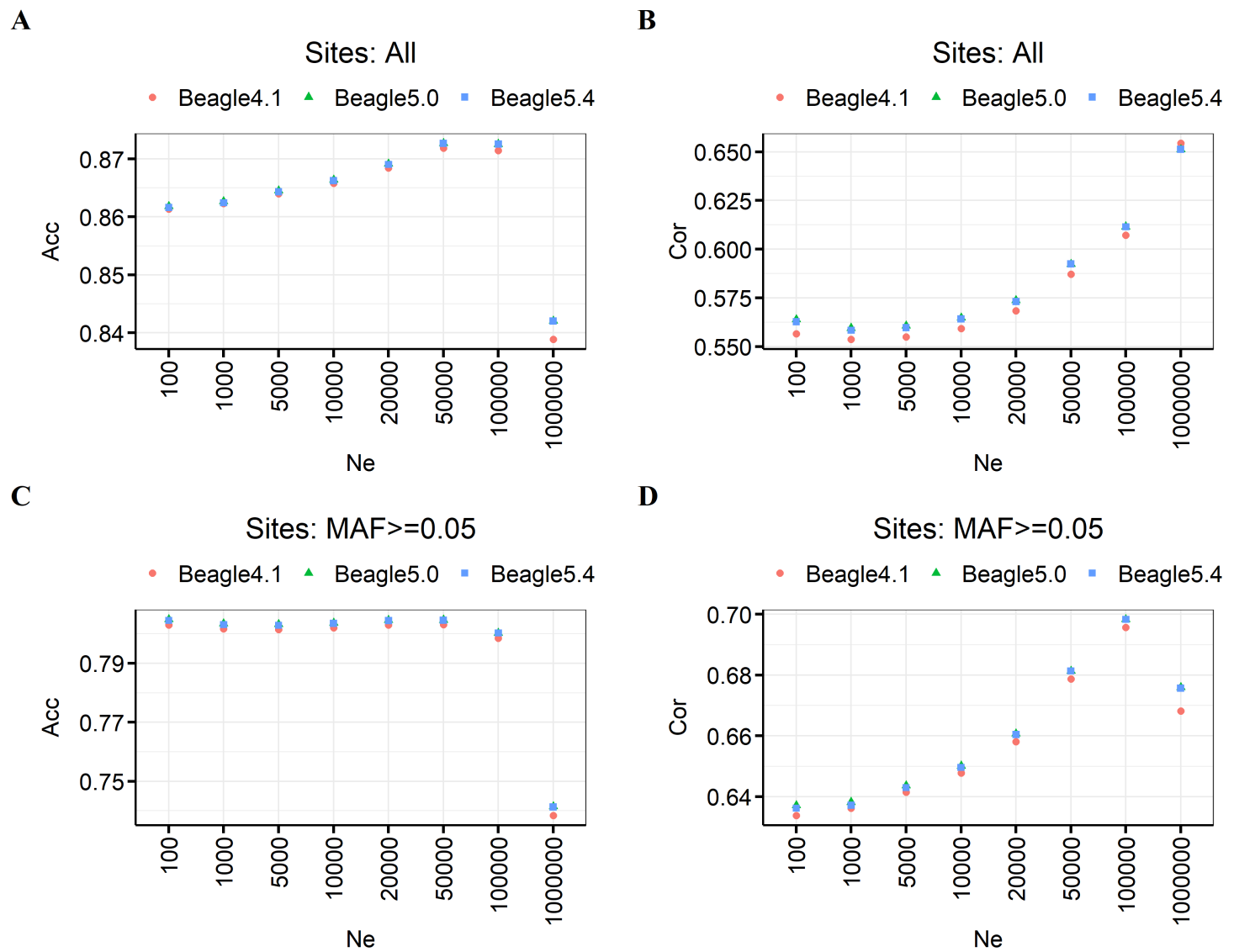


**Supplementary Figure 1.** Accuracy of imputation for the three imputation software, with a range of effective population size (Ne) sets in pigs. (A) Imputation accuracy measured by the genotype concordance (Acc) (B) Imputation accuracy measured by the correlation (Cor) (C), (D) correspond to (A) and (B) with the sites with minor allele frequency no less than 0.05.


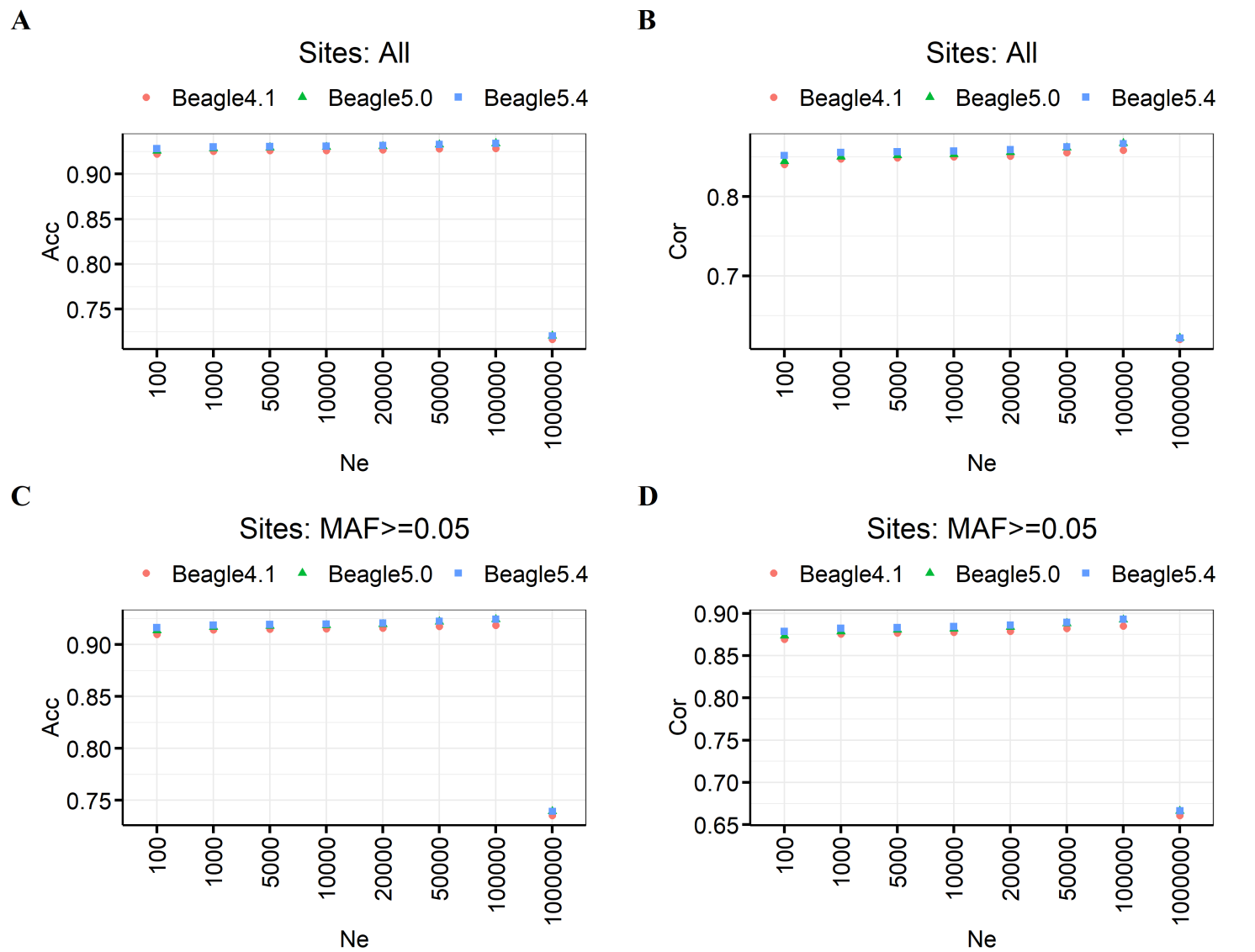


**Supplementary Figure 2.** Accuracy of imputation for the three imputation software with a range of effective population size (Ne) sets in chicken. (A) Imputation accuracy measured by the genotype concordance (Acc) (B) Imputation accuracy measured by the correlation (Cor) (C), (D) correspond to (A) and (B) with remove the sites with minor allele frequency less than 0.05.


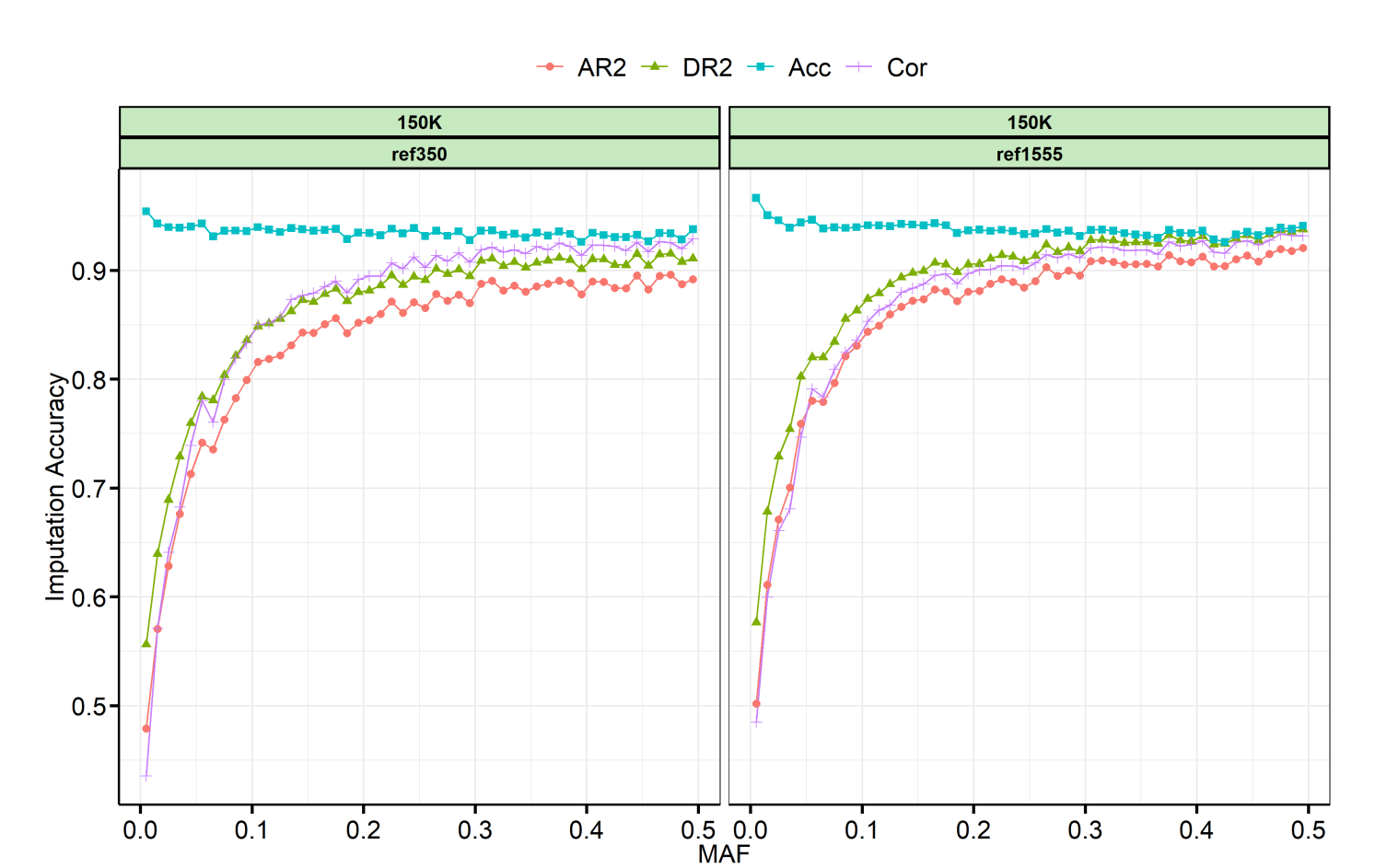


**Supplementary Figure 3.** Imputation accuracy by minor allele frequency (MAF) class. This imputation performed from 150K to WGS with ref350 or ref1555 using beagle 4.1 with Ne=100,000. The SNPs were divided into bins of 0.01 per increment according to their MAF. AR2, Allelic R-Squared; DR2, Dosage R-Squared; Acc, Genotype concordance; Cor: Correlation.


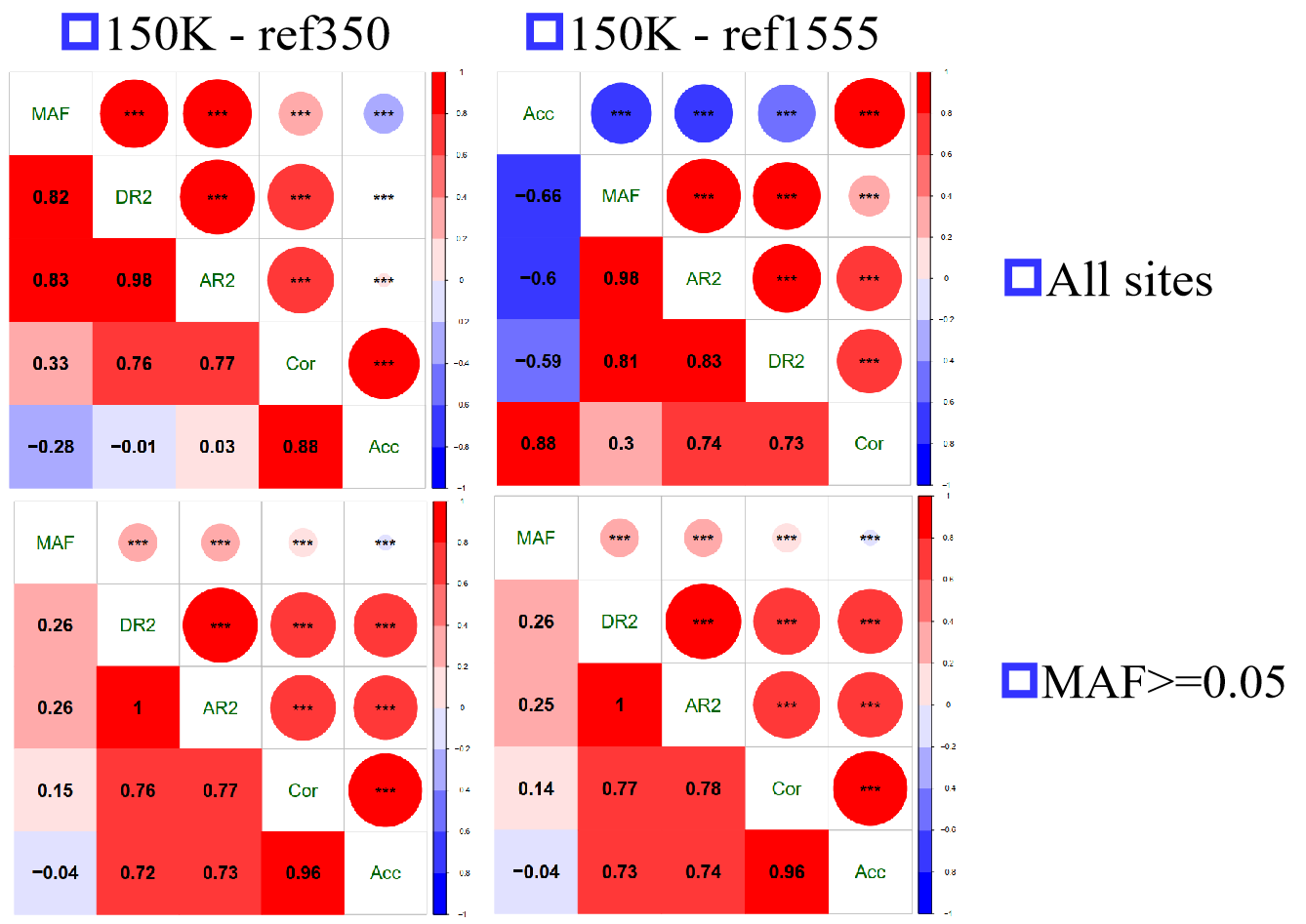


**Supplementary Figure 4.** The spearman correlation of the four measures of imputation accuracy and minor allele frequency (MAF) among each other. (A) All sites (B) The sites with minor allele frequency no less than 0.05.

## Supplementary Tables

**Supplementary Table 1.** The breed composition of all 1682 sequenced animals in RUN5 of the 1000 Bull Genome Project.

| **Class** | **Breed** | **Number** |
| --- | --- | --- |
| **Taurus** | Holstein | 450 |
|  | Fleckvieh | 145 |
|  | Angus | 141 |
|  | BrownSwiss | 105 |
|  | Simmental | 74 |
|  | Jersey | 66 |
|  | DanishRed | 44 |
|  | SimmentalFleckviehPezzatarossa | 43 |
|  | Gelbvieh | 41 |
|  | Charolais | 39 |
|  | Hereford | 37 |
|  | GuelphComposite | 33 |
|  | AlbertaComposite | 31 |
|  | Limousin | 31 |
|  | BeefBooster | 29 |
|  | Montbeliarde | 28 |
|  | FinnishAyrshire | 25 |
|  | Normande | 24 |
|  | NorwegianRed | 24 |
|  | Guernsey | 20 |
|  | HolsteinCharolais | 18 |
|  | OrigBraunviehBrownSwiss | 18 |
|  | AngusRed | 16 |
|  | SwedishRed | 16 |
|  | SimmentalDairy | 12 |
|  | BelgianBlue | 10 |
|  | Korean | 9 |
|  | Marchigiana | 8 |
|  | OrigBraunvieh | 8 |
|  | JapanNative | 8 |
|  | Angler | 5 |
|  | Piedmontese | 5 |
|  | SimmentalHolstein | 5 |
|  | HerefordPolled | 4 |
|  | Hinterwalder | 3 |
|  | SimmentalAngusRed | 3 |
|  | Vorderwalder | 3 |
|  | Eringer | 2 |
|  | JerseyLimousine | 2 |
|  | Romagnola | 2 |
|  | ScotishHighland | 2 |
|  | Stabilizer | 2 |
|  | TyroleanGrey | 2 |
|  | NA | 2 |
|  | BeltedGalloway | 1 |
|  | Galloway | 1 |
|  | GelbviehLimousine | 1 |
|  | LimousinHolstein | 1 |
|  | PiedmonteseNormande | 1 |
|  | Salers | 1 |
|  | SimmentalAngus | 1 |
| **Indicus** | UgandaAdmixed | 26 |
|  | IranAdmixed | 9 |
|  | Brahman | 7 |
|  | Gir | 6 |
|  | Nelore | 5 |
| **Chinese yellow cattle** | Dabieshan | 2 |
|  | Dehong | 2 |
|  | Dengchuan | 2 |
|  | Fujian | 2 |
|  | Guanling | 2 |
|  | Hasake | 2 |
|  | Liping | 2 |
|  | Luxi | 2 |
|  | Menggu | 2 |
|  | Nanyang | 2 |
|  | Qinchuan | 2 |
|  | Wenling | 2 |
|  | Yanbian | 2 |
|  | Xizang | 1 |

**Supplementary Table 2.** The sample information of 409 pigs.

| GVMSampleID | SampleName | Breed | Class |
| --- | --- | --- | --- |
| ssc.s1 | 0F0202 | Erhualian | Asia |
| ssc.s2 | 1729 | Duroc | Europe |
| ssc.s3 | 1735 | Duroc | Europe |
| ssc.s4 | 1795 | Duroc | Europe |
| ssc.s5 | 1933 | Duroc | Europe |
| ssc.s6 | 1964 | Duroc | Europe |
| ssc.s7 | 24-64 | Duroc | Europe |
| ssc.s8 | 24-78 | Duroc | Europe |
| ssc.s9 | 25-24 | Duroc | Europe |
| ssc.s10 | 25-78 | Duroc | Europe |
| ssc.s11 | 25-80 | Duroc | Europe |
| ssc.s12 | 26-23 | Duroc | Europe |
| ssc.s13 | 26-66 | Duroc | Europe |
| ssc.s14 | 27-20 | Duroc | Europe |
| ssc.s15 | 27-78 | Duroc | Europe |
| ssc.s16 | 27-81 | Duroc | Europe |
| ssc.s17 | 9268 | Yucatan_miniature_pig | Europe |
| ssc.s18 | 9273 | Yucatan_miniature_pig | Europe |
| ssc.s19 | 9274 | Yucatan_miniature_pig | Europe |
| ssc.s20 | 9281 | Yucatan_miniature_pig | Europe |
| ssc.s21 | 9293 | Yucatan_miniature_pig | Europe |
| ssc.s22 | AS01F01 | Europe | Europe |
| ssc.s23 | AS01F09 | Europe | Europe |
| ssc.s24 | B12 | Tibetan | Asia |
| ssc.s25 | B40 | Tibetan | Asia |
| ssc.s26 | BB01M47 | Europe | Europe |
| ssc.s27 | BD009U01 | Europe_Wild | Europe |
| ssc.s28 | BK01F10 | Europe | Europe |
| ssc.s29 | BK01M20 | Europe | Europe |
| ssc.s30 | BMX0001 | Bamaxiang | Asia |
| ssc.s31 | BMX0005 | Bamaxiang | Asia |
| ssc.s32 | BMX0012 | Bamaxiang | Asia |
| ssc.s33 | BMX0013 | Bamaxiang | Asia |
| ssc.s34 | BMX0014 | Bamaxiang | Asia |
| ssc.s35 | BMX0015 | Bamaxiang | Asia |
| ssc.s36 | BS01F10 | Europe | Europe |
| ssc.s37 | BS01F35 | Europe | Europe |
| ssc.s38 | B_1 | Berkshire | Europe |
| ssc.s39 | B_10 | Berkshire | Europe |
| ssc.s40 | B_2 | Berkshire | Europe |
| ssc.s41 | B_3 | Berkshire | Europe |
| ssc.s42 | B_4 | Berkshire | Europe |
| ssc.s43 | B_5 | Berkshire | Europe |
| ssc.s44 | B_6 | Berkshire | Europe |
| ssc.s45 | B_7 | Berkshire | Europe |
| ssc.s46 | B_8 | Berkshire | Europe |
| ssc.s47 | B_9 | Berkshire | Europe |
| ssc.s48 | CA01F14 | Europe | Europe |
| ssc.s49 | CM01F17 | Europe | Europe |
| ssc.s50 | CM01F18 | Europe | Europe |
| ssc.s51 | CS01F02 | Europe | Europe |
| ssc.s52 | CT01F13 | Europe | Europe |
| ssc.s53 | CT01M12 | Europe | Europe |
| ssc.s54 | DAA8330 | Duroc | Europe |
| ssc.s55 | DAA8623 | Duroc | Europe |
| ssc.s56 | DAA9119 | Duroc | Europe |
| ssc.s57 | DAA9736 | Duroc | Europe |
| ssc.s58 | DAA9738 | Duroc | Europe |
| ssc.s59 | DQZ26 | Tibetan | Asia |
| ssc.s60 | DU22M01 | Europe | Europe |
| ssc.s61 | DU22M02 | Europe | Europe |
| ssc.s62 | DU23M03 | Europe | Europe |
| ssc.s63 | DU23M04 | Europe | Europe |
| ssc.s64 | ER_CS0234 | Erhualian | Asia |
| ssc.s65 | ER_CS3544 | Erhualian | Asia |
| ssc.s66 | GO01F04 | Europe | Europe |
| ssc.s67 | GO01F23 | Europe | Europe |
| ssc.s68 | HTDE12 | Hetao | Asia |
| ssc.s69 | HTDE125 | Hetao | Asia |
| ssc.s70 | HTDE328 | Hetao | Asia |
| ssc.s71 | HTDE70 | Hetao | Asia |
| ssc.s72 | JI01U08 | Asian | Asia |
| ssc.s73 | JI01U10 | Asian | Asia |
| ssc.s74 | JQ01U02 | Asian | Asia |
| ssc.s75 | JQ01U03 | Asian | Asia |
| ssc.s76 | JQ01U08 | Asian | Asia |
| ssc.s77 | KL1 | Yorkshire | Europe |
| ssc.s78 | KL2 | Yorkshire | Europe |
| ssc.s79 | KL3 | Yorkshire | Europe |
| ssc.s80 | KL4 | Yorkshire | Europe |
| ssc.s81 | KL5 | Yorkshire | Europe |
| ssc.s82 | KL6 | Yorkshire | Europe |
| ssc.s83 | KL7 | Yorkshire | Europe |
| ssc.s84 | KL8 | Yorkshire | Europe |
| ssc.s85 | LB01F49 | Europe | Europe |
| ssc.s86 | LCP | Enshi_black | Asia |
| ssc.s87 | LE01F25 | Europe | Europe |
| ssc.s88 | LR21M03_Landrace | Europe | Europe |
| ssc.s89 | LR24F08_Landrace | Europe | Europe |
| ssc.s90 | LR30F03_Landrace | Europe | Europe |
| ssc.s91 | LR30F04_Landrace | Europe | Europe |
| ssc.s92 | LS01F04 | Europe | Europe |
| ssc.s93 | LSP01U16 | Europe | Asia |
| ssc.s94 | LSP01U18 | Europe | Asia |
| ssc.s95 | LTZ2011 | LitangZang | Asia |
| ssc.s96 | LTZ2016 | LitangZang | Asia |
| ssc.s97 | LUC201 | Luchuan | Asia |
| ssc.s98 | LUC2016 | Luchuan | Asia |
| ssc.s99 | LUC202 | Luchuan | Asia |
| ssc.s100 | LUC206 | Luchuan | Asia |
| ssc.s101 | LUC207 | Luchuan | Asia |
| ssc.s102 | LW22M04_LargeWhite | Europe | Europe |
| ssc.s103 | LW37F01 | Europe | Europe |
| ssc.s104 | LW38M02 | Europe | Europe |
| ssc.s105 | LWH0F | Laiwu | Asia |
| ssc.s106 | LWH0M | Laiwu | Asia |
| ssc.s107 | LWH1183 | Laiwu | Asia |
| ssc.s108 | LWH13 | Laiwu | Asia |
| ssc.s109 | LWH879 | Laiwu | Asia |
| ssc.s110 | MA01F18 | Europe | Europe |
| ssc.s111 | MA01F20 | Europe | Europe |
| ssc.s112 | MS20M03 | Asian | Asia |
| ssc.s113 | MS20M05 | Asian | Asia |
| ssc.s114 | MS20U10 | Asian | Asia |
| ssc.s115 | MS20U11 | Asian | Asia |
| ssc.s116 | MS20U13 | Asian | Asia |
| ssc.s117 | MS21M01 | Asian | Asia |
| ssc.s118 | MS21M05 | Asian | Asia |
| ssc.s119 | MS21M07 | Asian | Asia |
| ssc.s120 | MS21M08 | Asian | Asia |
| ssc.s121 | MW01F29 | Europe | Europe |
| ssc.s122 | MW01F33 | Europe | Europe |
| ssc.s123 | MZhu3126 | Min | Asia |
| ssc.s124 | MZhu3134 | Min | Asia |
| ssc.s125 | MZhu3312 | Min | Asia |
| ssc.s126 | NCYZ0013 | Wild_boar | Asia |
| ssc.s127 | NI01U07 | Europe | Europe |
| ssc.s128 | NS01F05 | Europe | Europe |
| ssc.s129 | PI21M20_Pietrain | Europe | Europe |
| ssc.s130 | Pietrain10 | Pietrain_breed | Europe |
| ssc.s131 | Pietrain11 | Pietrain_breed | Europe |
| ssc.s132 | Pietrain2 | Pietrain_breed | Europe |
| ssc.s133 | Pietrain3 | Pietrain_breed | Europe |
| ssc.s134 | Pietrain4 | Pietrain_breed | Europe |
| ssc.s135 | Pietrain5 | Pietrain_breed | Europe |
| ssc.s136 | Pietrain6 | Pietrain_breed | Europe |
| ssc.s137 | Pietrain7 | Pietrain_breed | Europe |
| ssc.s138 | Pietrain8 | Pietrain_breed | Europe |
| ssc.s139 | Pietrain9 | Pietrain_breed | Europe |
| ssc.s140 | R05 | Tibetan | Asia |
| ssc.s141 | R06 | Tibetan | Asia |
| ssc.s142 | R11 | Tibetan | Asia |
| ssc.s143 | RE01F51 | Europe | Europe |
| ssc.s144 | SVSV01U01 | Outgroup | Sus |
| ssc.s145 | SYWB103 | Wild_boar | Asia |
| ssc.s146 | SYWB205 | Wild_boar | Asia |
| ssc.s147 | S_1 | Yorkshire | Europe |
| ssc.s148 | S_10 | Landrace | Europe |
| ssc.s149 | S_11 | Landrace | Europe |
| ssc.s150 | S_12 | Landrace | Europe |
| ssc.s151 | S_13 | Landrace | Europe |
| ssc.s152 | S_14 | Landrace | Europe |
| ssc.s153 | S_15 | Landrace | Europe |
| ssc.s154 | S_16 | Landrace | Europe |
| ssc.s155 | S_17 | Landrace | Europe |
| ssc.s156 | S_18 | Landrace | Europe |
| ssc.s157 | S_19 | Landrace | Europe |
| ssc.s158 | S_2 | Yorkshire | Europe |
| ssc.s159 | S_20 | Landrace | Europe |
| ssc.s160 | S_3 | Yorkshire | Europe |
| ssc.s161 | S_4 | Yorkshire | Europe |
| ssc.s162 | S_5 | Yorkshire | Europe |
| ssc.s163 | S_6 | Yorkshire | Europe |
| ssc.s164 | S_7 | Landrace | Europe |
| ssc.s165 | S_8 | Landrace | Europe |
| ssc.s166 | S_9 | Landrace | Europe |
| ssc.s167 | TA01F19 | Europe | Europe |
| ssc.s168 | TA01M06 | Europe | Europe |
| ssc.s169 | W1 | Korean_wild | Asia |
| ssc.s170 | W10 | Korean_wild | Asia |
| ssc.s171 | W2 | Korean_wild | Asia |
| ssc.s172 | W3 | Korean_wild | Asia |
| ssc.s173 | W4 | Korean_wild | Asia |
| ssc.s174 | W5 | Korean_wild | Asia |
| ssc.s175 | W6 | Korean_wild | Asia |
| ssc.s176 | W7 | Korean_wild | Asia |
| ssc.s177 | W8 | Korean_wild | Asia |
| ssc.s178 | W9 | Korean_wild | Asia |
| ssc.s179 | WB20U02 | Asian_Wild | Asia |
| ssc.s180 | WB21F05 | Europe_Wild | Europe |
| ssc.s181 | WB21F10 | Europe_Wild | Europe |
| ssc.s182 | WB22F01 | Europe_Wild | Europe |
| ssc.s183 | WB22F02 | Europe_Wild | Europe |
| ssc.s184 | WB25U11_WildBoar | Europe_Wild | Europe |
| ssc.s185 | WB28F31 | Europe_Wild | Europe |
| ssc.s186 | WB28M39 | Europe_Wild | Europe |
| ssc.s187 | WB29U04 | Asian_Wild | Asia |
| ssc.s188 | WB29U12 | Asian_Wild | Asia |
| ssc.s189 | WB29U13 | Asian_Wild | Asia |
| ssc.s190 | WB29U16 | Asian_Wild | Asia |
| ssc.s191 | WB30U01 | Asian_Wild | Asia |
| ssc.s192 | WB30U08 | Asian_Wild | Asia |
| ssc.s193 | WB30U09 | Asian_Wild | Asia |
| ssc.s194 | WB31F05 | Europe_Wild | Europe |
| ssc.s195 | WB31M09 | Europe_Wild | Europe |
| ssc.s196 | WB32F07 | Europe_Wild | Europe |
| ssc.s197 | WB32U05 | Europe_Wild | Europe |
| ssc.s198 | WB33U04 | Europe_Wild | Europe |
| ssc.s199 | WB33U05 | Europe_Wild | Europe |
| ssc.s200 | WB42M09 | Europe_Wild | Europe |
| ssc.s201 | WB44U06 | Europe_Wild | Europe |
| ssc.s202 | WB44U07 | Europe_Wild | Europe |
| ssc.s203 | WB72U01 | Europe_Wild | Europe |
| ssc.s204 | WS01U03 | Asian | Asia |
| ssc.s205 | WZS149 | Wuzhishan | Asia |
| ssc.s206 | WZS809 | Wuzhishan | Asia |
| ssc.s207 | WZS889 | Wuzhishan | Asia |
| ssc.s208 | WZS947 | Wuzhishan | Asia |
| ssc.s209 | WZS955 | Wuzhishan | Asia |
| ssc.s210 | WZS973 | Wuzhishan | Asia |
| ssc.s211 | XI01U03_Xiang | Asian | Asia |
| ssc.s212 | XI01U04 | Asian | Asia |
| ssc.s213 | YTL | Enshi_black | Asia |
| ssc.s214 | ZA01U02 | Asian | Asia |
| ssc.s215 | ZBZ | Enshi_black | Asia |
| ssc.s216 | ZJWB01 | Wild_boar | Asia |
| ssc.s217 | ZJWB02 | Wild_boar | Asia |
| ssc.s218 | cebifrons1 | Sus_cebifrons | Sus |
| ssc.s219 | cebifrons2 | Sus_cebifrons | Sus |
| ssc.s220 | cebifrons3 | Sus_cebifrons | Sus |
| ssc.s221 | cebifrons4 | Sus_cebifrons | Sus |
| ssc.s222 | cebifrons5 | Sus_cebifrons | Sus |
| ssc.s223 | cebifrons6 | Sus_cebifrons | Sus |
| ssc.s224 | cebifrons7 | Sus_cebifrons | Sus |
| ssc.s225 | pig31 | Yorkshire | Europe |
| ssc.s226 | pig32 | Yorkshire | Europe |
| ssc.s227 | sample1 | Korean | Asia |
| ssc.s228 | sample10 | Jeju_black_pig | Asia |
| ssc.s229 | sample11 | Jeju_black_pig | Asia |
| ssc.s230 | sample12 | Jeju_black_pig | Asia |
| ssc.s231 | sample13 | Jeju_black_pig | Asia |
| ssc.s232 | sample14 | Jeju_black_pig | Asia |
| ssc.s233 | sample2 | Korean | Asia |
| ssc.s234 | sample3 | Korean | Asia |
| ssc.s235 | sample4 | Korean | Asia |
| ssc.s236 | sample5 | Korean | Asia |
| ssc.s237 | sample6 | Korean | Asia |
| ssc.s238 | sample7 | Jeju_black_pig | Asia |
| ssc.s239 | sample8 | Jeju_black_pig | Asia |
| ssc.s240 | sample9 | Jeju_black_pig | Asia |
| ssc.s241 | w09-085 | Yucatan_miniature_pig | Europe |
| ssc.s242 | w10-001 | Yucatan_miniature_pig | Europe |
| ssc.s243 | w10-075 | Yucatan_miniature_pig | Europe |
| ssc.s244 | w10-082 | Yucatan_miniature_pig | Europe |
| ssc.s245 | w10-088 | Yucatan_miniature_pig | Europe |
| ssc.s246 | w11-012 | Yucatan_miniature_pig | Europe |
| ssc.s247 | w11-059 | Yucatan_miniature_pig | Europe |
| ssc.s248 | SME001 | Diannan_small_ear | Asia |
| ssc.s249 | SME002 | Diannan_small_ear | Asia |
| ssc.s250 | SME003 | Diannan_small_ear | Asia |
| ssc.s251 | SME004 | Diannan_small_ear | Asia |
| ssc.s252 | SME005 | Diannan_small_ear | Asia |
| ssc.s253 | SME006 | Diannan_small_ear | Asia |
| ssc.s254 | SME007 | Diannan_small_ear | Asia |
| ssc.s255 | SME008 | Diannan_small_ear | Asia |
| ssc.s256 | SME009 | Diannan_small_ear | Asia |
| ssc.s257 | SME010 | Diannan_small_ear | Asia |
| ssc.s258 | SME011 | Diannan_small_ear | Asia |
| ssc.s259 | SME012 | Diannan_small_ear | Asia |
| ssc.s260 | SME013 | Diannan_small_ear | Asia |
| ssc.s261 | SME014 | Diannan_small_ear | Asia |
| ssc.s262 | SME015 | Diannan_small_ear | Asia |
| ssc.s263 | SME016 | Diannan_small_ear | Asia |
| ssc.s264 | SME017 | Diannan_small_ear | Asia |
| ssc.s265 | SME018 | Diannan_small_ear | Asia |
| ssc.s266 | SME019 | Diannan_small_ear | Asia |
| ssc.s267 | SME020 | Diannan_small_ear | Asia |
| ssc.s268 | SME021 | Diannan_small_ear | Asia |
| ssc.s269 | SME022 | Diannan_small_ear | Asia |
| ssc.s270 | SME023 | Diannan_small_ear | Asia |
| ssc.s271 | SME024 | Diannan_small_ear | Asia |
| ssc.s272 | SME025 | Diannan_small_ear | Asia |
| ssc.s273 | SME026 | Diannan_small_ear | Asia |
| ssc.s274 | SME027 | Diannan_small_ear | Asia |
| ssc.s275 | SME028 | Diannan_small_ear | Asia |
| ssc.s276 | SME029 | Diannan_small_ear | Asia |
| ssc.s277 | SME030 | Diannan_small_ear | Asia |
| ssc.s278 | DU23M01_Duroc | Duroc | Europe |
| ssc.s279 | DU23M02_Duroc | Duroc | Europe |
| ssc.s280 | DU23M03_Duroc | Duroc | Europe |
| ssc.s281 | DU23M04_Duroc | Duroc | Europe |
| ssc.s282 | HA20U01_Hampshire | Hampshire | Europe |
| ssc.s283 | HA20U02_Hampshire | Hampshire | Europe |
| ssc.s284 | JQ01U02_Jiangquhai | Jiangquhai | Asia |
| ssc.s285 | LR21M03 | Landrace | Europe |
| ssc.s286 | LR24F01 | Landrace | Europe |
| ssc.s287 | LR24F08 | Landrace | Europe |
| ssc.s288 | LR30F02 | Landrace | Europe |
| ssc.s289 | LR30F03 | Landrace | Europe |
| ssc.s290 | LW22F01 | Large_White | Europe |
| ssc.s291 | LW22F02 | Large_White | Europe |
| ssc.s292 | LW22F03 | Large_White | Europe |
| ssc.s293 | LW22F04 | Large_White | Europe |
| ssc.s294 | LW22F06 | Large_White | Europe |
| ssc.s295 | LW22F07 | Large_White | Europe |
| ssc.s296 | LW22M04 | Large_White | Europe |
| ssc.s297 | LW22M07 | Large_White | Europe |
| ssc.s298 | LW36F01 | Large_White | Europe |
| ssc.s299 | LW36F02 | Large_White | Europe |
| ssc.s300 | LW36F03 | Large_White | Europe |
| ssc.s301 | LW36F04 | Large_White | Europe |
| ssc.s302 | LW36F05 | Large_White | Europe |
| ssc.s303 | LW36F06 | Large_White | Europe |
| ssc.s304 | MS20U10_Meishan | Meishan | Asia |
| ssc.s305 | MS20U11_Meishan | Meishan | Asia |
| ssc.s306 | MS21M07_Meishan | Meishan | Asia |
| ssc.s307 | MS21M14_Meishan | Meishan | Asia |
| ssc.s308 | PI21F02 | Pietrain | Europe |
| ssc.s309 | PI21F06 | Pietrain | Europe |
| ssc.s310 | PI21M17 | Pietrain | Europe |
| ssc.s311 | PI21M20 | Pietrain | Europe |
| ssc.s312 | PI21M21 | Pietrain | Europe |
| ssc.s313 | WB20U02_Japan | Wild_Boar_Japan | Asia |
| ssc.s314 | WB21F05_Netherlands | Europe_Wild | Europe |
| ssc.s315 | WB21M03_Netherlands | Europe_Wild | Europe |
| ssc.s316 | WB22F01_NL | Europe_Wild | Europe |
| ssc.s317 | WB22F02_NL | Europe_Wild | Europe |
| ssc.s318 | WB25U11 | Europe_Wild | Europe |
| ssc.s319 | WB26M09_Malcantone | Chinese_Wild | Europe |
| ssc.s320 | WB29U04_SChina | Chinese_Wild | Asia |
| ssc.s321 | WB29U12_SChina | Chinese_Wild | Asia |
| ssc.s322 | WB30U01_NChina | Chinese_Wild | Asia |
| ssc.s323 | WB30U08_NChina | Chinese_Wild | Asia |
| ssc.s324 | XI01U03 | Xiang | Asia |
| ssc.s325 | XI01U04_Xiang | Xiang | Asia |
| ssc.s326 | BGI_CWB_NCYZ0010 | Chinese_Wild | Asia |
| ssc.s327 | BGI_LUC_LUC208 | Luchuan | Asia |
| ssc.s328 | BGI_TT_MLZ7 | Tibetan | Asia |
| ssc.s329 | BGI_TT_MLZ14 | Tibetan | Asia |
| ssc.s330 | BGI_TT_MLZ30 | Tibetan | Asia |
| ssc.s331 | BGI_TT_B26 | Tibetan | Asia |
| ssc.s332 | BGI_YNT_DQZ12 | Tibetan | Asia |
| ssc.s333 | BGI_YNT_DQZ4 | Tibetan | Asia |
| ssc.s334 | BGI_YNT_DQZ19 | Tibetan | Asia |
| ssc.s335 | BGI_YNT_DQZ24 | Tibetan | Asia |
| ssc.s336 | BGI_YNT_DQZ25 | Tibetan | Asia |
| ssc.s337 | BGI_SCT_LTZ201 | Tibetan | Asia |
| ssc.s338 | BGI_SCT_LTZ203 | Tibetan | Asia |
| ssc.s339 | BGI_SCT_LTZ206 | Tibetan | Asia |
| ssc.s340 | BGI_SCT_LTZ208 | Tibetan | Asia |
| ssc.s341 | BGI_GST_R02 | Tibetan | Asia |
| ssc.s342 | BGI_HTDE_HTDE201 | Hetao | Asia |
| ssc.s343 | BGI_HTDE_HTDE314 | Hetao | Asia |
| ssc.s344 | BGI_MIN_MZ-304-07 | Min | Asia |
| ssc.s345 | BGI_MIN_MZ-307-00 | Min | Asia |
| ssc.s346 | BGI_MIN_MZhu3252 | Min | Asia |
| ssc.s347 | BGI_LWH_LWH181 | Laiwu | Asia |
| ssc.s348 | BGI_EHL_0F0090 | Erhualian | Asia |
| ssc.s349 | BGI_EHL_0F0094 | Erhualian | Asia |
| ssc.s350 | SRS387185 | Tibetan | Asia |
| ssc.s351 | SRS387186 | Tibetan | Asia |
| ssc.s352 | SRS387190 | Tibetan | Asia |
| ssc.s353 | SRS387202 | Tibetan | Asia |
| ssc.s354 | SRS387204 | Tibetan | Asia |
| ssc.s355 | Tibetan_pig-6 | Tibetan | Asia |
| ssc.s356 | Tibetan_pig-8 | Tibetan | Asia |
| ssc.s357 | Tibetan_pig-9 | Tibetan | Asia |
| ssc.s358 | Tibetan_pig-11 | Tibetan | Asia |
| ssc.s359 | Tibetan_pig-12 | Tibetan | Asia |
| ssc.s360 | Tibetan_pig-13 | Tibetan | Asia |
| ssc.s361 | Tibetan_pig-14 | Tibetan | Asia |
| ssc.s362 | Tibetan_pig-15 | Tibetan | Asia |
| ssc.s363 | Tibetan_pig-16 | Tibetan | Asia |
| ssc.s364 | Tibetan_pig-17 | Tibetan | Asia |
| ssc.s365 | Tibetan_pig-18 | Tibetan | Asia |
| ssc.s366 | Tibetan_pig-19 | Tibetan | Asia |
| ssc.s367 | Tibetan_pig-20 | Tibetan | Asia |
| ssc.s368 | Tibetan_pig-21 | Tibetan | Asia |
| ssc.s369 | Tibetan_pig-22 | Tibetan | Asia |
| ssc.s370 | Tibetan_pig-23 | Tibetan | Asia |
| ssc.s371 | Tibetan_pig-24 | Tibetan | Asia |
| ssc.s372 | Tibetan_pig-25 | Tibetan | Asia |
| ssc.s373 | Tibetan_pig-26 | Tibetan | Asia |
| ssc.s374 | SRS387278 | Tibetan | Asia |
| ssc.s375 | SRS387280 | Tibetan | Asia |
| ssc.s376 | SRS387282 | Tibetan | Asia |
| ssc.s377 | SRS387284 | Tibetan | Asia |
| ssc.s378 | Penzhou_pig-1 | Penzhou | Asia |
| ssc.s379 | Penzhou_pig-3 | Penzhou | Asia |
| ssc.s380 | Wujin_pig-1 | Wujin | Asia |
| ssc.s381 | Wujin_pig-2 | Wujin | Asia |
| ssc.s382 | Wujin_pig-3 | Wujin | Asia |
| ssc.s383 | Ya'nan_pig-1 | Ya'nan | Asia |
| ssc.s384 | Ya'nan_pig-2 | Ya'nan | Asia |
| ssc.s385 | Ya'nan_pig-3 | Ya'nan | Asia |
| ssc.s386 | Neijiang_pig-1 | Neijiang | Asia |
| ssc.s387 | Neijiang_pig-2 | Neijiang | Asia |
| ssc.s388 | Neijiang_pig-3 | Neijiang | Asia |
| ssc.s389 | Jinhua_pig-1 | Jinhua | Asia |
| ssc.s390 | Jinhua_pig-2 | Jinhua | Asia |
| ssc.s391 | Jinhua_pig-3 | Jinhua | Asia |
| ssc.s392 | Wild_boar-1 | Chinese_Wild | Asia |
| ssc.s393 | Wild_boar-2 | Chinese_Wild | Asia |
| ssc.s394 | Wild_boar-3 | Chinese_Wild | Asia |
| ssc.s395 | WB29U14 | Chinese_Wild | Asia |
| ssc.s396 | WB21F03 | Europe_Wild | Europe |
| ssc.s397 | WB22M03 | Europe_Wild | Europe |
| ssc.s398 | WB21F04 | Europe_Wild | Europe |
| ssc.s399 | WB21M05 | Europe_Wild | Europe |
| ssc.s400 | WB22F03 | Europe_Wild | Europe |
| ssc.s401 | WB22F04 | Europe_Wild | Europe |
| ssc.s402 | WB26M09 | Asian_Wild | Asia |
| ssc.s403 | OG-1 | Sumatran_wild_boar | Sus |
| ssc.s404 | OG-2 | Bearded | Sus |
| ssc.s405 | OG-3 | Sumatran_wild_boar | Sus |
| ssc.s406 | OG-4 | Warthog | Sus |
| ssc.s407 | OG-5 | Sus_cebifrons | Sus |
| ssc.s408 | OG-6 | Sus_celebensis | Sus |
| ssc.s409 | OG-7 | Sus_verrucosus | Sus |

**Supplementary Table 3.** The sample information of 335 chickens.

| ID | Type | Breed | Class |
| --- | --- | --- | --- |
| G114 | WGS | yellow-feather_dwarf_broiler | YFDB |
| G117 | WGS | yellow-feather_dwarf_broiler | YFDB |
| G128 | WGS | yellow-feather_dwarf_broiler | YFDB |
| G133 | WGS | yellow-feather_dwarf_broiler | YFDB |
| G14 | WGS | yellow-feather_dwarf_broiler | YFDB |
| G172 | WGS | yellow-feather_dwarf_broiler | YFDB |
| G20 | WGS | yellow-feather_dwarf_broiler | YFDB |
| G23 | WGS | yellow-feather_dwarf_broiler | YFDB |
| G246 | WGS | yellow-feather_dwarf_broiler | YFDB |
| G275 | WGS | yellow-feather_dwarf_broiler | YFDB |
| G512 | WGS | yellow-feather_dwarf_broiler | YFDB |
| G544 | WGS | yellow-feather_dwarf_broiler | YFDB |
| G546 | WGS | yellow-feather_dwarf_broiler | YFDB |
| G561 | WGS | yellow-feather_dwarf_broiler | YFDB |
| G664 | WGS | yellow-feather_dwarf_broiler | YFDB |
| G669 | WGS | yellow-feather_dwarf_broiler | YFDB |
| G678 | WGS | yellow-feather_dwarf_broiler | YFDB |
| G702 | WGS | yellow-feather_dwarf_broiler | YFDB |
| G707 | WGS | yellow-feather_dwarf_broiler | YFDB |
| G726 | WGS | yellow-feather_dwarf_broiler | YFDB |
| G727 | WGS | yellow-feather_dwarf_broiler | YFDB |
| G734 | WGS | yellow-feather_dwarf_broiler | YFDB |
| G740 | WGS | yellow-feather_dwarf_broiler | YFDB |
| G747 | WGS | yellow-feather_dwarf_broiler | YFDB |
| DRR089959 | PRJDB4092 | GJFj | GJF |
| DRR089960 | PRJDB4092 | GJFj | GJF |
| DRR089961 | PRJDB4092 | GJFm | GJF |
| DRR089962 | PRJDB4092 | GJFm | GJF |
| DRR089963 | PRJDB4092 | GJFm | GJF |
| DRR089964 | PRJDB4092 | GJFm | GJF |
| DRR089965 | PRJDB4092 | GJFm | GJF |
| DRR089966 | PRJDB4092 | GJFm | GJF |
| DRR089967 | PRJDB4092 | GJFm | GJF |
| DRR089968 | PRJDB4092 | RJFj | RJF |
| DRR089969 | PRJDB4092 | RJFj | RJF |
| DRR089970 | PRJDB4092 | RJFj | RJF |
| DRR089971 | PRJDB4092 | RJFs | RJF |
| DRR089972 | PRJDB4092 | RJFs | RJF |
| DRR089973 | PRJDB4092 | Sumatra | Else |
| DRR089974 | PRJDB4092 | Sumatra | Else |
| DRR089975 | PRJDB4092 | Sumatra | Else |
| DRR089976 | PRJDB4092 | Sumatra | Else |
| DRR089977 | PRJDB4092 | Sumatra | Else |
| DRR089978 | PRJDB4092 | Black_Sumatra | Else |
| DRR089979 | PRJDB4092 | Black_Sumatra | Else |
| DRR089980 | PRJDB4092 | Black_Sumatra | Else |
| DRR089981 | PRJDB4092 | Black_Sumatra | Else |
| DRR089982 | PRJDB4092 | Black_Sumatra | Else |
| DRR089983 | PRJDB4092 | Black_Sumatra | Else |
| DRR089984 | PRJDB4092 | Black_Sumatra | Else |
| DRR089985 | PRJDB4092 | Black_Sumatra | Else |
| DRR089986 | PRJDB4092 | Black_Sumatra | Else |
| DRR089987 | PRJDB4092 | Black_Sumatra | Else |
| DRR089988 | PRJDB4092 | Kedu_Hitam | Else |
| DRR089989 | PRJDB4092 | Kedu_Hitam | Else |
| DRR089990 | PRJDB4092 | Kedu_Hitam | Else |
| DRR089991 | PRJDB4092 | Kedu_Hitam | Else |
| DRR089992 | PRJDB4092 | Kedu_Hitam | Else |
| DRR089993 | PRJDB4092 | Kedu_Hitam | Else |
| DRR089994 | PRJDB4092 | Kedu_Hitam | Else |
| DRR089995 | PRJDB4092 | Kedu_Hitam | Else |
| DRR089996 | PRJDB4092 | Kedu_Hitam | Else |
| DRR089997 | PRJDB4092 | Kedu_Hitam | Else |
| DRR089998 | PRJDB4092 | Black_Java | Else |
| DRR089999 | PRJDB4092 | Black_Java | Else |
| DRR090000 | PRJDB4092 | Black_Java | Else |
| DRR090001 | PRJDB4092 | Black_Java | Else |
| DRR090002 | PRJDB4092 | Black_Java | Else |
| DRR090003 | PRJDB4092 | Black_Java | Else |
| DRR090004 | PRJDB4092 | Black_Java | Else |
| DRR090005 | PRJDB4092 | Black_Java | Else |
| DRR090006 | PRJDB4092 | Black_Java | Else |
| DRR090007 | PRJDB4092 | Black_Java | Else |
| DRR090008 | PRJDB4092 | WL-G | Else |
| DRR090009 | PRJDB4092 | WL-G | Else |
| DRR090010 | PRJDB4092 | WL-G | Else |
| SRR851160 | PRJNA202483 | Silkie | Else |
| SRR867742 | PRJNA202483 | Silkie | Else |
| SRR867743 | PRJNA202483 | Silkie | Else |
| SRR867744 | PRJNA202483 | Silkie | Else |
| SRR867746 | PRJNA202483 | Silkie | Else |
| SRR867747 | PRJNA202483 | Silkie | Else |
| SRR867748 | PRJNA202483 | Taiwanese_native_chicken_L2 | Else |
| SRR867749 | PRJNA202483 | Taiwanese_native_chicken_L2 | Else |
| SRR867750 | PRJNA202483 | Taiwanese_native_chicken_L2 | Else |
| SRR867767 | PRJNA202483 | Taiwanese_native_chicken_L2 | Else |
| SRR867768 | PRJNA202483 | Taiwanese_native_chicken_L2 | Else |
| SRR1174199 | PRJNA232548 | Beijing_You | Else |
| SRR1174201 | PRJNA232548 | Beijing_You | Else |
| SRR1174880 | PRJNA232548 | Cornish | Else |
| SRR1176892 | PRJNA232548 | Dongxiang | Else |
| SRR1181639 | PRJNA232548 | Luxi_Game | Else |
| SRR1182456 | PRJNA232548 | Rhode_Island_Red | Else |
| SRR1182854 | PRJNA232548 | RJF | RJF |
| SRR1184215 | PRJNA232548 | Shouguang | Else |
| SRR1184459 | PRJNA232548 | Shouguang | Else |
| SRR1185309 | PRJNA232548 | Silkie | Else |
| SRR1185319 | PRJNA232548 | Silkie | Else |
| SRR1185937 | PRJNA232548 | Tibetan | Else |
| SRR1185941 | PRJNA232548 | Tibetan | Else |
| SRR1185947 | PRJNA232548 | Wenchang | Else |
| SRR1185948 | PRJNA232548 | Wenchang | Else |
| SRR1185952 | PRJNA232548 | White_Leghorn | Else |
| SRR1185953 | PRJNA232548 | White_Leghorn | Else |
| SRR1186050 | PRJNA232548 | White_Plymouth_Rock | Else |
| SRR1186051 | PRJNA232548 | White_Plymouth_Rock | Else |
| SRR1217491 | PRJNA241474 | Tibetan | Else |
| SRR1217492 | PRJNA241474 | Tibetan | Else |
| SRR1217493 | PRJNA241474 | Tibetan | Else |
| SRR1217494 | PRJNA241474 | Tibetan | Else |
| SRR1217495 | PRJNA241474 | Tibetan | Else |
| SRR1217496 | PRJNA241474 | Tibetan | Else |
| SRR1217497 | PRJNA241474 | Tibetan | Else |
| SRR1217498 | PRJNA241474 | Tibetan | Else |
| SRR1217499 | PRJNA241474 | Tibetan | Else |
| SRR1217500 | PRJNA241474 | Tibetan | Else |
| SRR1217501 | PRJNA241474 | Tibetan | Else |
| SRR1217502 | PRJNA241474 | Tibetan | Else |
| SRR1217503 | PRJNA241474 | Tibetan | Else |
| SRR1217504 | PRJNA241474 | Tibetan | Else |
| SRR1217505 | PRJNA241474 | Tibetan | Else |
| SRR1217506 | PRJNA241474 | Tibetan | Else |
| SRR1217507 | PRJNA241474 | Tibetan | Else |
| SRR1217508 | PRJNA241474 | Tibetan | Else |
| SRR1217509 | PRJNA241474 | fighting_chicken | Else |
| SRR1217510 | PRJNA241474 | fighting_chicken | Else |
| SRR1217511 | PRJNA241474 | fighting_chicken | Else |
| SRR1217512 | PRJNA241474 | fighting_chicken | Else |
| SRR1217513 | PRJNA241474 | fighting_chicken | Else |
| SRR1217514 | PRJNA241474 | fighting_chicken | Else |
| SRR1217515 | PRJNA241474 | fighting_chicken | Else |
| SRR1217516 | PRJNA241474 | fighting_chicken | Else |
| SRR1217517 | PRJNA241474 | fighting_chicken | Else |
| SRR1217519 | PRJNA241474 | fighting_chicken | Else |
| SRR1217520 | PRJNA241474 | fighting_chicken | Else |
| SRR1217521 | PRJNA241474 | fighting_chicken | Else |
| SRR1217522 | PRJNA241474 | fighting_chicken | Else |
| SRR1217523 | PRJNA241474 | fighting_chicken | Else |
| SRR1217524 | PRJNA241474 | RJF | RJF |
| SRR1217526 | PRJNA241474 | RJF | RJF |
| SRR1217527 | PRJNA241474 | RJF | RJF |
| SRR1217528 | PRJNA241474 | RJF | RJF |
| SRR1217529 | PRJNA241474 | RJF | RJF |
| SRR1217530 | PRJNA241474 | RJF | RJF |
| SRR1217531 | PRJNA241474 | RJF | RJF |
| SRR1217532 | PRJNA241474 | RJF | RJF |
| SRR1217533 | PRJNA241474 | RJF | RJF |
| SRR1217534 | PRJNA241474 | RJF | RJF |
| SRR1217535 | PRJNA241474 | native_chicken | Else |
| SRR1217536 | PRJNA241474 | native_chicken | Else |
| SRR1217537 | PRJNA241474 | native_chicken | Else |
| SRR1217538 | PRJNA241474 | native_chicken | Else |
| SRR1217539 | PRJNA241474 | native_chicken | Else |
| SRR1217540 | PRJNA241474 | native_chicken | Else |
| SRR1217541 | PRJNA241474 | native_chicken | Else |
| SRR1217542 | PRJNA241474 | native_chicken | Else |
| SRR1217543 | PRJNA241474 | native_chicken | Else |
| SRR1217544 | PRJNA241474 | native_chicken | Else |
| SRR1217545 | PRJNA241474 | native_chicken | Else |
| SRR1217546 | PRJNA241474 | native_chicken | Else |
| SRR1217547 | PRJNA241474 | native_chicken | Else |
| SRR1217548 | PRJNA241474 | native_chicken | Else |
| SRR1217549 | PRJNA241474 | native_chicken | Else |
| SRR1217550 | PRJNA241474 | native_chicken | Else |
| SRR1217551 | PRJNA241474 | native_chicken | Else |
| SRR1217552 | PRJNA241474 | native_chicken | Else |
| SRR1217553 | PRJNA241474 | native_chicken | Else |
| SRR1217554 | PRJNA241474 | commercial_chicken | Else |
| SRR1217555 | PRJNA241474 | Tibetan | Else |
| SRR1217556 | PRJNA241474 | Tibetan | Else |
| SRR1217557 | PRJNA241474 | Tibetan | Else |
| SRR1217558 | PRJNA241474 | Tibetan | Else |
| SRR1217559 | PRJNA241474 | Tibetan | Else |
| SRR1217560 | PRJNA241474 | Tibetan | Else |
| SRR1217561 | PRJNA241474 | Tibetan | Else |
| SRR1217562 | PRJNA241474 | Tibetan | Else |
| SRR1291152 | PRJNA247952 | cross_2meat_type_chicken_lines | Else |
| SRR1291153 | PRJNA247952 | cross_2meat_type_chicken_lines | Else |
| SRR1291155 | PRJNA247952 | cross_2meat_type_chicken_lines | Else |
| SRR1291158 | PRJNA247952 | cross_2meat_type_chicken_lines | Else |
| SRR1291159 | PRJNA247952 | cross_2meat_type_chicken_lines | Else |
| SRR1291160 | PRJNA247952 | cross_2meat_type_chicken_lines | Else |
| SRR1291161 | PRJNA247952 | cross_2meat_type_chicken_lines | Else |
| SRR1291162 | PRJNA247952 | cross_2meat_type_chicken_lines | Else |
| SRR1291164 | PRJNA247952 | cross_2meat_type_chicken_lines | Else |
| SRR1291165 | PRJNA247952 | cross_2meat_type_chicken_lines | Else |
| SRR1559302 | PRJNA247952 | cross_2meat_type_chicken_lines | Else |
| SRR1559303 | PRJNA247952 | cross_2meat_type_chicken_lines | Else |
| SRR1559304 | PRJNA247952 | cross_2meat_type_chicken_lines | Else |
| SRR1559305 | PRJNA247952 | cross_2meat_type_chicken_lines | Else |
| SRR1559306 | PRJNA247952 | cross_2meat_type_chicken_lines | Else |
| SRR1559307 | PRJNA247952 | cross_2meat_type_chicken_lines | Else |
| SRR1559308 | PRJNA247952 | cross_2meat_type_chicken_lines | Else |
| SRR1559309 | PRJNA247952 | cross_2meat_type_chicken_lines | Else |
| SRR1559310 | PRJNA247952 | cross_2meat_type_chicken_lines | Else |
| SRR1559311 | PRJNA247952 | cross_2meat_type_chicken_lines | Else |
| SRR1559312 | PRJNA247952 | cross_2meat_type_chicken_lines | Else |
| SRR1320249 | PRJNA251505 | cross_2meat_type_chicken_lines | Else |
| SRR1320250 | PRJNA251505 | cross_2meat_type_chicken_lines | Else |
| SRR1320251 | PRJNA251505 | cross_2meat_type_chicken_lines | Else |
| SRR1320252 | PRJNA251505 | cross_2meat_type_chicken_lines | Else |
| SRR1320253 | PRJNA251505 | cross_2meat_type_chicken_lines | Else |
| SRR1320254 | PRJNA251505 | cross_2meat_type_chicken_lines | Else |
| SRR1320255 | PRJNA251505 | cross_2meat_type_chicken_lines | Else |
| SRR1320256 | PRJNA251505 | cross_2meat_type_chicken_lines | Else |
| SRR1320257 | PRJNA251505 | cross_2meat_type_chicken_lines | Else |
| SRR1320269 | PRJNA251505 | cross_2meat_type_chicken_lines | Else |
| SRR1320299 | PRJNA251505 | cross_2meat_type_chicken_lines | Else |
| SRR1320320 | PRJNA251505 | cross_2meat_type_chicken_lines | Else |
| SRR1320535 | PRJNA251505 | cross_2meat_type_chicken_lines | Else |
| SRR1320578 | PRJNA251505 | cross_2meat_type_chicken_lines | Else |
| SRR1320604 | PRJNA251505 | cross_2meat_type_chicken_lines | Else |
| SRR1320648 | PRJNA251505 | cross_2meat_type_chicken_lines | Else |
| SRR1320649 | PRJNA251505 | cross_2meat_type_chicken_lines | Else |
| SRR1320653 | PRJNA251505 | cross_2meat_type_chicken_lines | Else |
| SRR1320758 | PRJNA251505 | cross_2meat_type_chicken_lines | Else |
| SRR1320760 | PRJNA251505 | cross_2meat_type_chicken_lines | Else |
| SRR1744074 | PRJNA271711 | 3GA_3LH_3KNC | Else |
| SRR1744075 | PRJNA271711 | 3GA_3LH_3KNC | Else |
| SRR1744077 | PRJNA271711 | 3GA_3LH_3KNC | Else |
| SRR1744078 | PRJNA271711 | 3GA_3LH_3KNC | Else |
| SRR1744079 | PRJNA271711 | 3GA_3LH_3KNC | Else |
| SRR1744080 | PRJNA271711 | 3GA_3LH_3KNC | Else |
| SRR1744081 | PRJNA271711 | 3GA_3LH_3KNC | Else |
| SRR1744082 | PRJNA271711 | 3GA_3LH_3KNC | Else |
| SRR1744083 | PRJNA271711 | 3GA_3LH_3KNC | Else |
| SRR2154196 | PRJNA292383 | Village | Else |
| SRR2154198 | PRJNA292383 | Village | Else |
| SRR2154199 | PRJNA292383 | Village | Else |
| SRR2154200 | PRJNA292383 | Village | Else |
| SRR2154201 | PRJNA292383 | Village | Else |
| SRR2154203 | PRJNA292383 | Village | Else |
| SRR2154224 | PRJNA292383 | Village | Else |
| SRR2154237 | PRJNA292383 | Village | Else |
| SRR2154242 | PRJNA292383 | Village | Else |
| SRR2154250 | PRJNA292383 | Village | Else |
| SRR2154298 | PRJNA292383 | Village | Else |
| SRR2154321 | PRJNA292383 | Village | Else |
| SRR3036337 | PRJNA306389 | Tibetan | Else |
| SRR3036360 | PRJNA306389 | Tibetan | Else |
| SRR3041115 | PRJNA306389 | Tibetan | Else |
| SRR3041116 | PRJNA306389 | Tibetan | Else |
| SRR3041121 | PRJNA306389 | Tibetan | Else |
| SRR3041122 | PRJNA306389 | Tibetan | Else |
| SRR3041123 | PRJNA306389 | Tibetan | Else |
| SRR3041124 | PRJNA306389 | Tibetan | Else |
| SRR3041125 | PRJNA306389 | Tibetan | Else |
| SRR3041126 | PRJNA306389 | Tibetan | Else |
| SRR3041127 | PRJNA306389 | Tibetan | Else |
| SRR3041128 | PRJNA306389 | Tibetan | Else |
| SRR3041129 | PRJNA306389 | Tibetan | Else |
| SRR3041130 | PRJNA306389 | Tibetan | Else |
| SRR3041131 | PRJNA306389 | Tibetan | Else |
| SRR3041132 | PRJNA306389 | Tibetan | Else |
| SRR3041133 | PRJNA306389 | Tibetan | Else |
| SRR3041134 | PRJNA306389 | Tibetan | Else |
| SRR3041135 | PRJNA306389 | Tibetan | Else |
| SRR3041136 | PRJNA306389 | Tibetan | Else |
| SRR3041137 | PRJNA306389 | Tibetan | Else |
| SRR3041138 | PRJNA306389 | Tibetan | Else |
| SRR3041364 | PRJNA306389 | Tibetan | Else |
| SRR3041409 | PRJNA306389 | Tibetan | Else |
| SRR3041410 | PRJNA306389 | Tibetan | Else |
| SRR3041411 | PRJNA306389 | Tibetan | Else |
| SRR3041412 | PRJNA306389 | Tibetan | Else |
| SRR3041413 | PRJNA306389 | Tibetan | Else |
| SRR3041414 | PRJNA306389 | Tibetan | Else |
| SRR3041415 | PRJNA306389 | Tibetan | Else |
| SRR3041416 | PRJNA306389 | Tibetan | Else |
| SRR3041417 | PRJNA306389 | Tibetan | Else |
| SRR3041418 | PRJNA306389 | Tibetan | Else |
| SRR3041419 | PRJNA306389 | Tibetan | Else |
| SRR3041420 | PRJNA306389 | Tibetan | Else |
| SRR3041421 | PRJNA306389 | Tibetan | Else |
| SRR3041422 | PRJNA306389 | Tibetan | Else |
| SRR3041423 | PRJNA306389 | Tibetan | Else |
| SRR3041425 | PRJNA306389 | Tibetan | Else |
| SRR3041426 | PRJNA306389 | Tibetan | Else |
| SRR3041427 | PRJNA306389 | Tibetan | Else |
| SRR3041428 | PRJNA306389 | Tibetan | Else |
| SRR3041433 | PRJNA306389 | Tibetan | Else |
| SRR3041434 | PRJNA306389 | Tibetan | Else |
| SRR3041435 | PRJNA306389 | Tibetan | Else |
| SRR3041436 | PRJNA306389 | Tibetan | Else |
| SRR3041437 | PRJNA306389 | Tibetan | Else |
| SRR3041438 | PRJNA306389 | Tibetan | Else |
| SRR3041439 | PRJNA306389 | Tibetan | Else |
| SRR3041440 | PRJNA306389 | Tibetan | Else |
| SRR3041441 | PRJNA306389 | Tibetan | Else |
| SRR3041442 | PRJNA306389 | Tibetan | Else |
| SRR3041443 | PRJNA306389 | Tibetan | Else |
| SRR3041444 | PRJNA306389 | Tibetan | Else |
| SRR3041445 | PRJNA306389 | Tibetan | Else |
| SRR3041446 | PRJNA306389 | Tibetan | Else |
| SRR3041447 | PRJNA306389 | Tibetan | Else |
| SRR3041448 | PRJNA306389 | Tibetan | Else |
| SRR3041449 | PRJNA306389 | Tibetan | Else |
| SRR3041450 | PRJNA306389 | Tibetan | Else |
| SRR3041451 | PRJNA306389 | Tibetan | Else |
| SRR3041452 | PRJNA306389 | Tibetan | Else |
| SRR3041453 | PRJNA306389 | Tibetan | Else |
| SRR3041454 | PRJNA306389 | Tibetan | Else |
| SRR3041455 | PRJNA306389 | Tibetan | Else |
| SRR3041456 | PRJNA306389 | Tibetan | Else |
| SRR3041457 | PRJNA306389 | Tibetan | Else |
| SRR3041458 | PRJNA306389 | Tibetan | Else |
| SRR3041504 | PRJNA306389 | Tibetan | Else |
| SRR3041573 | PRJNA306389 | Tibetan | Else |
| SRR3041620 | PRJNA306389 | Tibetan | Else |
| SRR3041692 | PRJNA306389 | Tibetan | Else |
| SRR3041713 | PRJNA306389 | Tibetan | Else |
| SRR3041781 | PRJNA306389 | Tibetan | Else |
| SRR3041923 | PRJNA306389 | Tibetan | Else |
| SRR3041924 | PRJNA306389 | Tibetan | Else |
| SRR3041925 | PRJNA306389 | Tibetan | Else |
| SRR3041926 | PRJNA306389 | Tibetan | Else |
| SRR4292973 | PRJNA344300 | Dagu | Else |
| SRR4293061 | PRJNA344300 | Emei_Black | Else |
| SRR4302052 | PRJNA344300 | Hetian | Else |
| SRR4302053 | PRJNA344300 | Luhua | Else |
| SRR4302054 | PRJNA344300 | Piao | Else |
| SRR4302055 | PRJNA344300 | Piao | Else |
| SRR4302056 | PRJNA344300 | Qianxiang | Else |
| SRR4302058 | PRJNA344300 | Taoyuan | Else |
| SRR4302059 | PRJNA344300 | Xianju | Else |
| SRR4302060 | PRJNA344300 | Emei_Black | Else |
| SRR4302061 | PRJNA344300 | Dagu | Else |
| SRR4302062 | PRJNA344300 | Longsheng | Else |
| SRR4302063 | PRJNA344300 | Nixi | Else |
| SRR4302064 | PRJNA344300 | Nixi | Else |
| SRR4302065 | PRJNA344300 | Nixi | Else |
| SRR4302066 | PRJNA344300 | Niya | Else |
| SRR4302067 | PRJNA344300 | Niya | Else |
| SRR4302068 | PRJNA344300 | Niya | Else |
| SRR4302069 | PRJNA344300 | Piao | Else |
